# Supplementary material for: Proline synthesis through PYCR1 is required to support cancer cell proliferation and survival in oxygen-limiting conditions
Source: Cell Rep. 2022 Feb 1;38(5):110320. doi: 10.1016/j.celrep.2022.110320 (PMC8822494; doi:10.1016/j.celrep.2022.110320)
Supplement: Document S1. Figures S1–S5 [file mmc1.pdf]

Cell Reports, Volume *38*

Supplemental information

Proline synthesis through PYCR1 is required to support cancer cell proliferation and survival in oxygen-limiting conditions

Rebecca L. Westbrook, Esther Bridges, Jennie Roberts, Cristina Escribano-Gonzalez, Katherine L. Eales, Lisa A. Vettore, Paul D. Walker, Elias Vera-Siguenza, Himani Rana, Federica Cuozzo, Kattri-Liis Eskla, Hans Vellama, Abeer Shaaban, Colin Nixon, Hendrik Luuk, Gareth G. Lavery, David J. Hodson, Adrian L. Harris, and Daniel A. Tennant

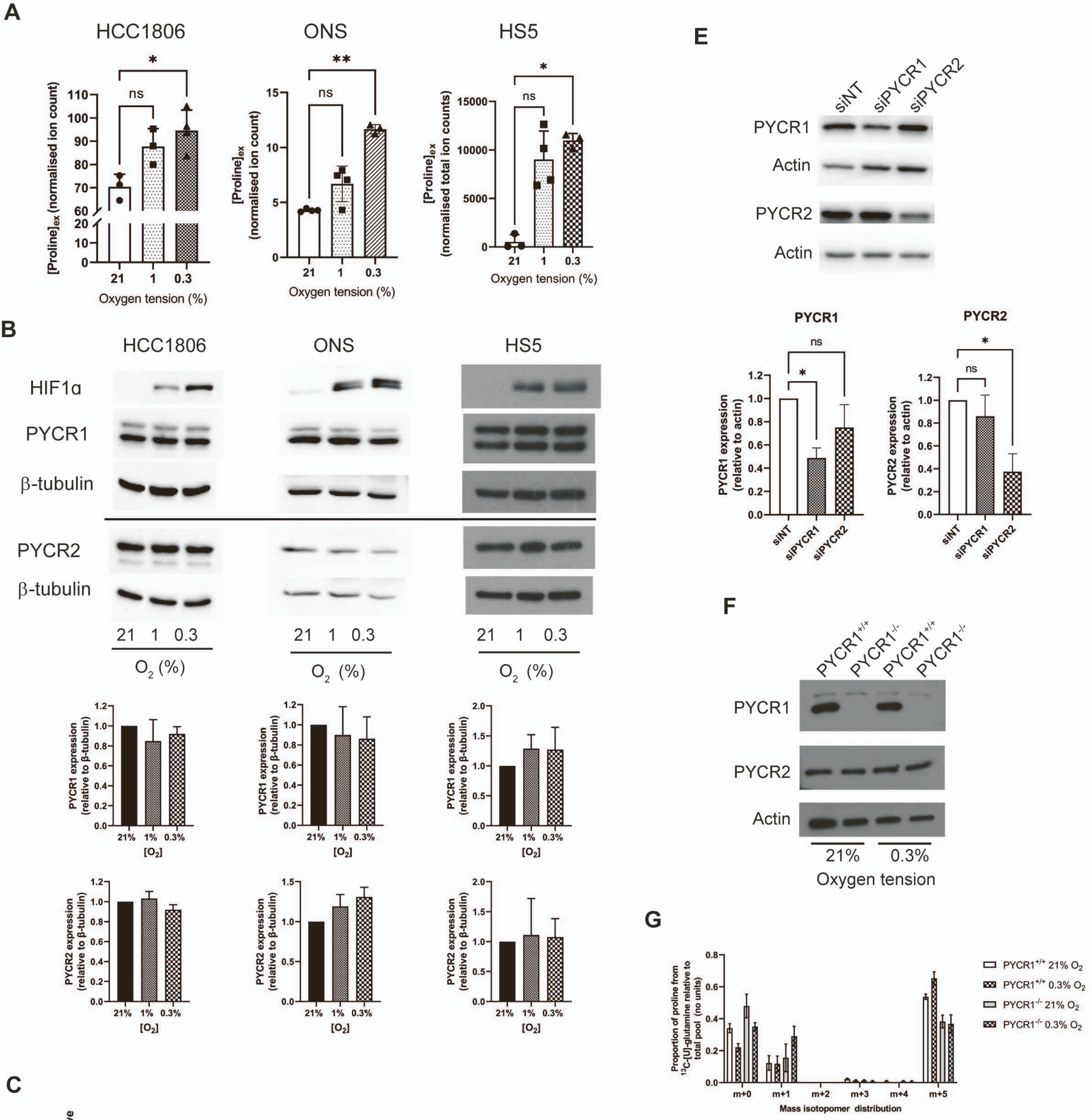

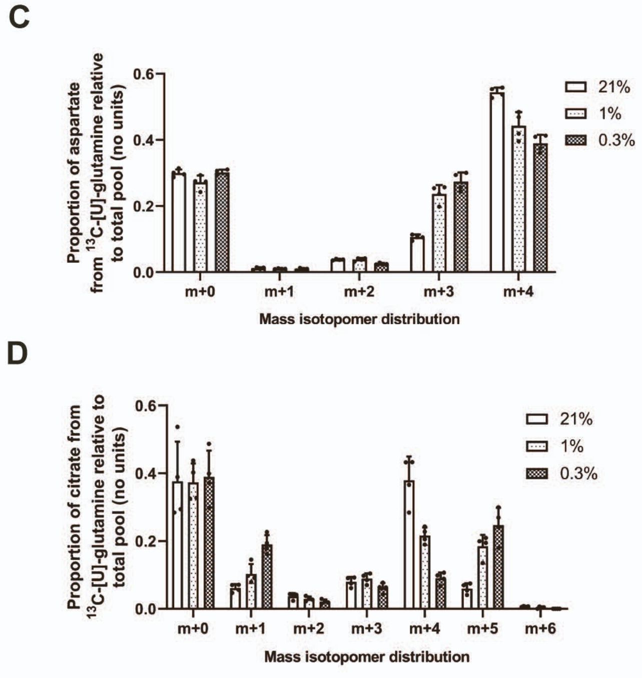

Supplementary Figure 1. Glutamine-derived proline export is increased in hypoxia without increased expression PYCR1 or PYCR2. Related to Figure 1. A: Extracellular proline is significantly increased in hypoxia (0.3% O_2) compared to normoxia (21% O_2) in the HCC1806, ONS and HS5 cell lines. *, p<0.05; **, p<0.01. **B:** Expression of PYCR1 and PYCR2 does not change with oxygen tension in HCC1806, ONS or HS5 cell lines. Densitometry shown is PYCR1 or 2 normalised to b-tubulin, SD of three independent blots shown. **C** and **D**: As oxygen decreases, the expected decrease in TCA cycle activity (m+4 aspartate [**C**] and citrate [**D**]) and relative increase in reductive carboxylation (m+5 citrate [**D**] and m+3 aspartate [**C**]) is observed in SUM159PT cells. **E:** Protein expression of PYCR1 and PYCR2 with siRNA knockdown of each isoform in SUM159PT cells. Actin as loading control. Densitometry shown below blots for PYCR1 and PYCR2 showing significant knockdown of the relevant isozyme. Statistical analysis shown is result of Kruskal-Wallis ANOVA with Dunn's post hoc test. *, p<0.05. **F:** Expression of PYCR1 and PYCR2 in the SUM159PT PYCR1$^{-/-}$ cell line in 21% and 0.3% oxygen. **G:** Mass isotopomer distribution of ^{13}C-[U]-glutamine incorporation into intracellular proline relative to the total pool in SUM159PT PYCR1$^{+/+}$ and PYCR1$^{-/-}$ cells. All metabolic data presented is mean of n=3 ± SD.

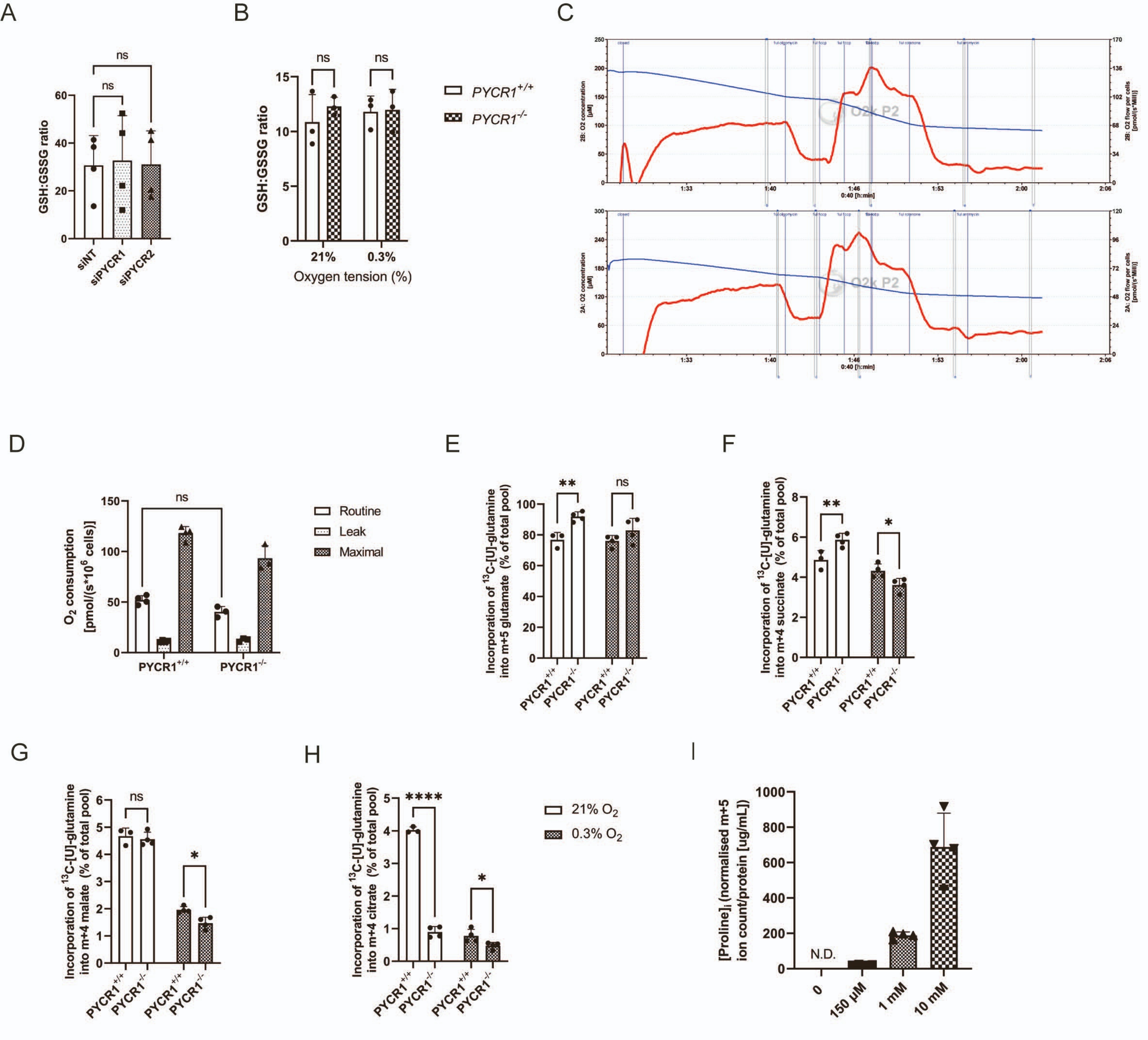

Supplementary Figure 2. Loss of PYCR1 in 2D cell culture. A: siPYCR1 or 2 does not change GSH:GSSG ratio in the SUM159PT cell line. Related to Figure 2. B: PYCR1$^{-/-}$ cells do not exhibit an alteration in the GSH:GSSG ratio in normoxia or hypoxia. **C:** Representative oxygraph traces of PYCR1$^{+/+}$ (top) and PYCR1$^{-/-}$ (bottom) cells. **D:** There is no significant difference in oxygen consumption in PYCR1$^{-/-}$ cells compared to PYCR1$^{+/+}$. Steady-state levels of glutamate (**E**), succinate (**F**), malate (**G**) and citrate (**H**). *, p<0.05; **, p<0.01; ****, p<0.0001. **I:** Abundance of intracellular ^{13}C-[U]-proline in SUM159PT cells incubated with increasing concentrations. All data presented as mean ± SD

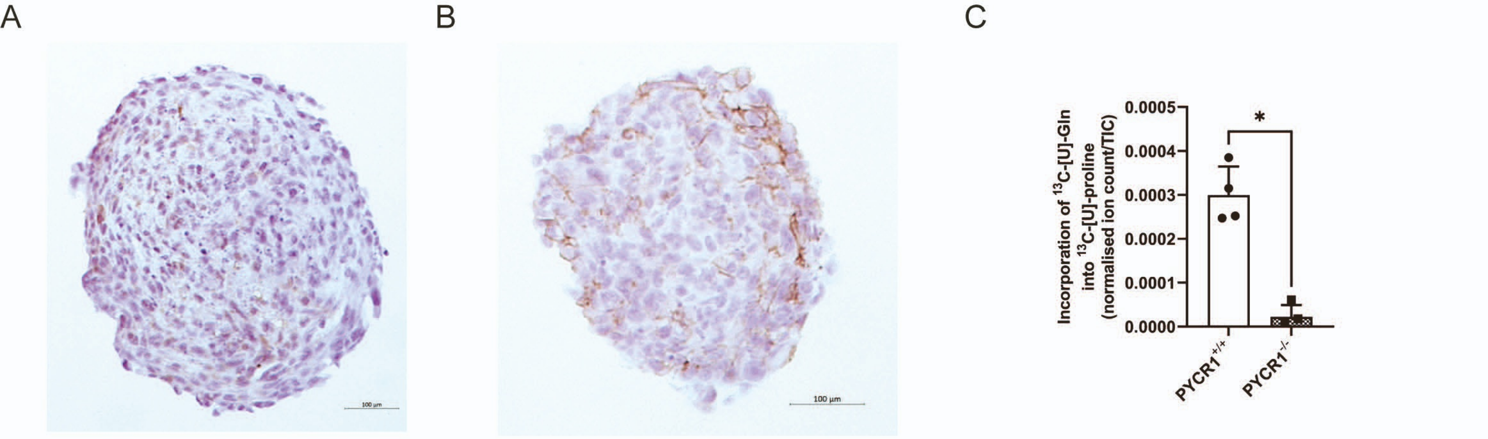

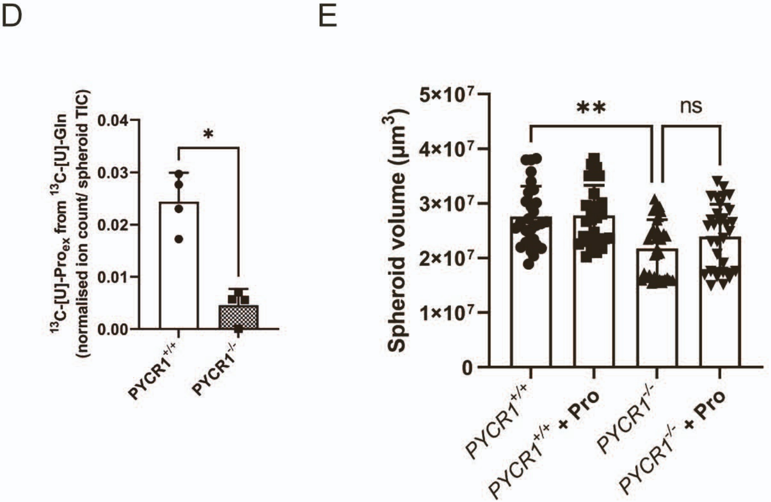

Supplementary Figure 3: Loss of PYCR1 in a 3D cell culture model. Related to Figure 3. A: Image of a representative SUM159PT spheroid stained with the hypoxia marker, pimonidazole, showing diffuse areas of hypoxia. **B:** Image showing staining of the hypoxia-induced glucose transporter, GLUT1 in SUM159PT spheroid. **C:** PYCR1$^{-/-}$ spheroids synthesise less ^{13}C-[U]-proline from ^{13}C-[U]-glutamine. *, p<0.05. This is reflected in reduced excretion of proline into the media (**D**), consistent with data from 2D culture. *, p<0.05. **E:** PYCR1$^{-/-}$ spheroid growth is not rescued by exogenous proline supplementation. All data presented as mean ± SD

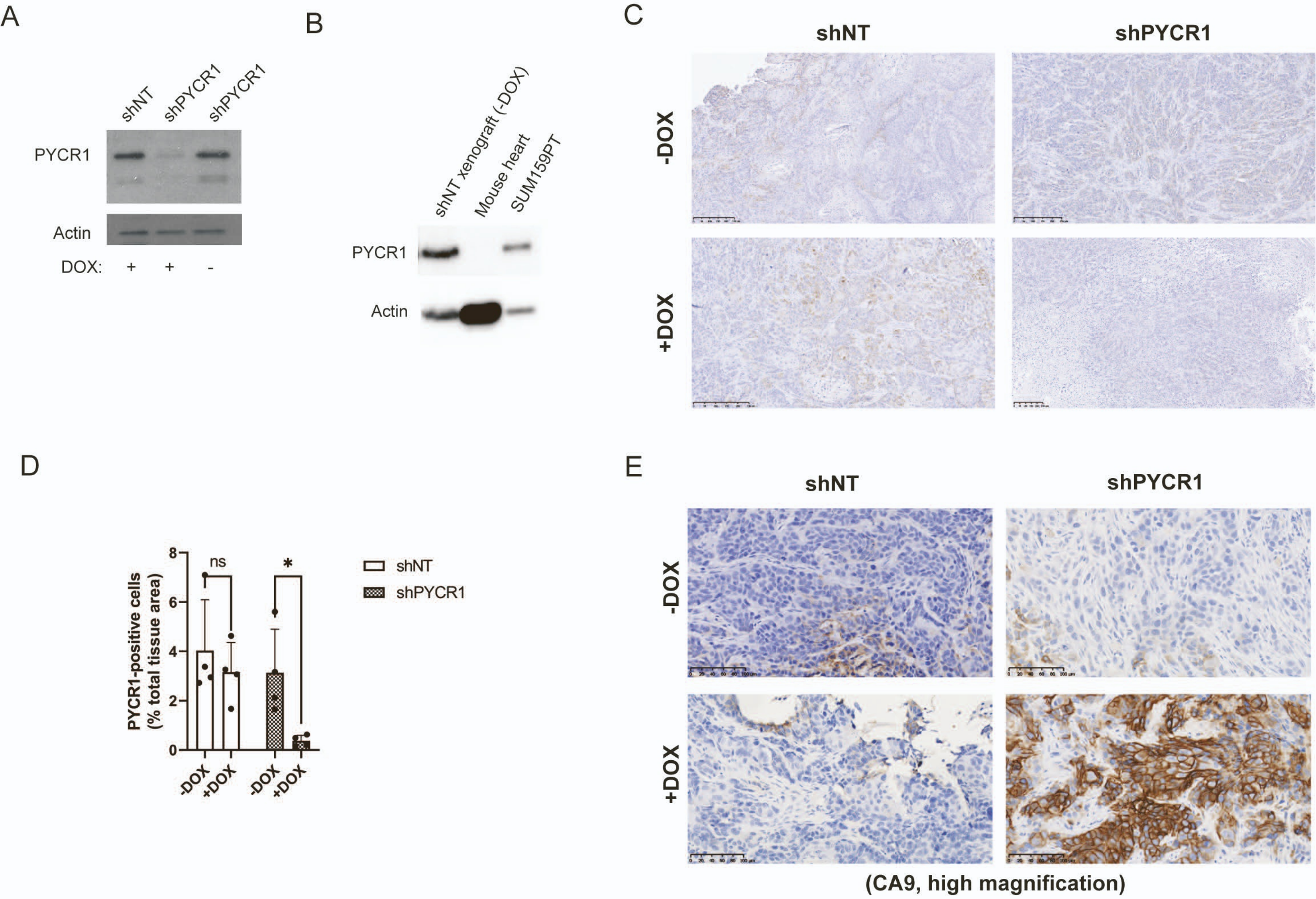

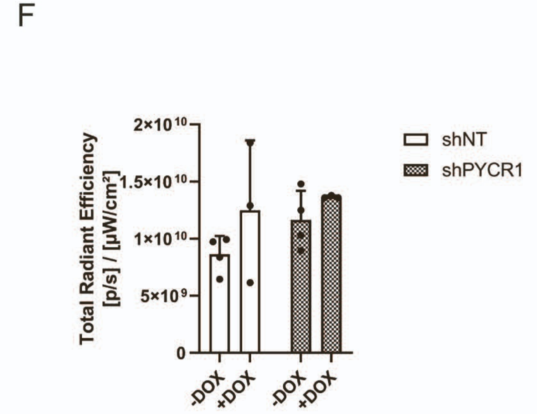

Supplementary Figure 4: Acute inducible PYCR1 knockdown in vivo xenografts. Related to Figure 4. A: Doxycycline elicits knockdown of PYCR1 in shPYCR1 cell model. **B:** Antibody against PYCR1 used to stain xenograft tissue (C) is specific for human protein. **C:** Representative images showing that PYCR1 expression in the tumour cells of the xenograft is reduced after DOX treatment. **D:** Quantification of PYCR1 staining in all tumours showing a reduction in PYCR1 expression after DOX treatment. *, $p<0.05$. **E:** Representative images at high magnification of CA9 staining in xenografts from the groups indicated, showing significantly increased hypoxia only in those tumours with shPYCR1+DOX. **F:** No significant differences in vascularity (measured through total radiant efficiency) were observed between groups (Figure 4). All quantitative data presented as mean ± SD

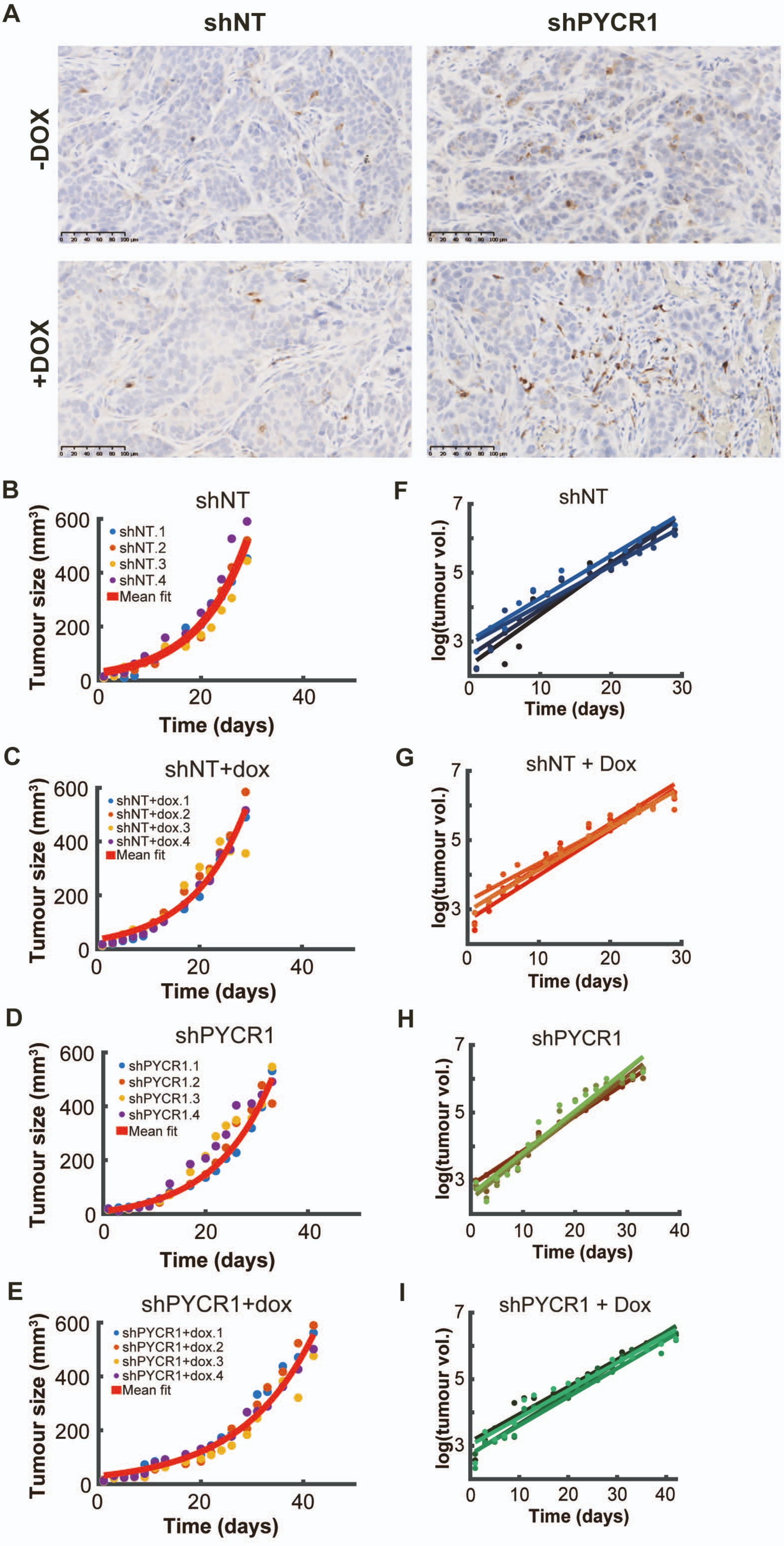

Supplementary Figure 5: Xenograft phenotype and xenograft growth curve fitting. Related to Figure 5. A: Representative higher magnification image of xenograft tumours stained for cleaved caspase-3. Scale bar = 100 mm. **B-E:** Exponential model fit of xenograft growth curves. **F-I:** Linear regression of log-transformed fitted curves B-E, allowing for appropriate statistical analysis (results shown in Figure 5F).
