# Supplementary material for: Proline synthesis through PYCR1 is required to support cancer cell proliferation and survival in oxygen-limiting conditions
Source: Cell Rep. 2022 Feb 1;38(5):110320. doi: 10.1016/j.celrep.2022.110320 (PMC8822494; doi:10.1016/j.celrep.2022.110320)
Supplement: Document S2. Article plus supplemental information [file mmc2.pdf]

Cell Reports

Article

Proline synthesis through PYCR1 is required to support cancer cell proliferation and survival in oxygen-limiting conditions

Graphical abstract

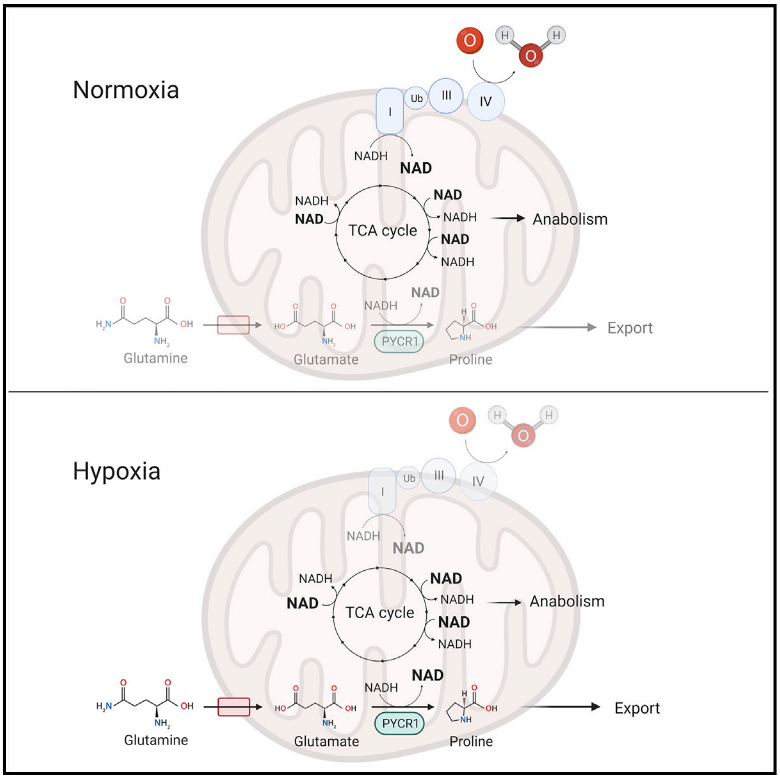

Highlights

- Proline synthesis through PYCR1 is increased in low-oxygen conditions
- PYCR1 activity in hypoxia supports TCA cycle function through NADH oxidation
- PYCR1 is required for maintenance of hypoxic tumor regions

Authors

Rebecca L. Westbrook, Esther Bridges, Jennie Roberts, ..., David J. Hodson, Adrian L. Harris, Daniel A. Tennant

Correspondence

d.tennant@bham.ac.uk

In brief

Westbrook et al. show that proline synthesis from glutamine through PYCR1 supports NADH oxidation when oxygen is limited. PYCR1 therefore represents an oxygen-independent means of regenerating NAD^+ in hypoxia, supporting continued oxidative TCA cycle activity. Loss of PYCR1 in tumors results in increased hypoxia, reduced cell viability, and necrosis.

Westbrook et al., 2022, Cell Reports 38, 110320
February 1, 2022 © 2022 The Authors.
https://doi.org/10.1016/j.celrep.2022.110320

CellPress

Cell Reports

CellPress
OPEN ACCESS

Article

Proline synthesis through PYCR1 is required to support cancer cell proliferation and survival in oxygen-limiting conditions

Rebecca L. Westbrook,[1] Esther Bridges,[2,3] Jennie Roberts,[1] Cristina Escribano-Gonzalez,[1] Katherine L. Eales,[1] Lisa A. Vettore,[1] Paul D. Walker,[1] Elias Vera-Siguenza,[1] Himani Rana,[1] Federica Cuozzo,[1] Kattri-Liis Eskla,[4,5] Hans Vellama,[4,5] Abeer Shaaban,[6] Colin Nixon,[7] Hendrik Luuk,[4,5] Gareth G. Lavery,[1,8] David J. Hodson,[1] Adrian L. Harris,[2] and Daniel A. Tennant[1,9,10,*]

[1]Institute of Metabolism and Systems Research, College of Medical and Dental Sciences, University of Birmingham, Birmingham B15 2TT, UK
[2]Hypoxia and Angiogenesis Group, Cancer Research UK Molecular Oncology Laboratories, Weatherall Institute of Molecular Medicine, Department of Oncology, University of Oxford, Oxford OX3 9DS, UK
[3]MRC Human Immunology Unit, MRC Weatherall Institute of Molecular Medicine, Department of Oncology, University of Oxford, Oxford OX3 9DS, UK
[4]Institute of Biomedicine and Translational Medicine, Department of Physiology, University of Tartu, Tartu, Estonia
[5]Centre of Excellence for Genomics and Translational Medicine, University of Tartu, Tartu, Estonia
[6]University Hospital Birmingham NHS Foundation Trust and Institute of Cancer and Genomic Sciences, College of Medical and Dental Sciences, University of Birmingham, Birmingham B15 2TT, UK
[7]Beatson Institute for Cancer Research, University of Glasgow, Switchback Road, Glasgow G61 1BD, UK
[8]Present address: Department of Biosciences, School of Science and Technology, Nottingham Trent University, Clifton Campus, Nottingham, UK
[9]Twitter: @tennantlab
[10]Lead contact
*Correspondence: d.tennant@bham.ac.uk
https://doi.org/10.1016/j.celrep.2022.110320

SUMMARY

The demands of cancer cell proliferation alongside an inadequate angiogenic response lead to insufficient oxygen availability in the tumor microenvironment. Within the mitochondria, oxygen is the major electron acceptor for NADH, with the result that the reducing potential produced through tricarboxylic acid (TCA) cycle activity and mitochondrial respiration are functionally linked. As the oxidizing activity of the TCA cycle is required for efficient synthesis of anabolic precursors, tumoral hypoxia could lead to a cessation of proliferation without another means of correcting the redox imbalance. We show that in hypoxic conditions, mitochondrial pyrroline 5-carboxylate reductase 1 (PYCR1) activity is increased, oxidizing NADH with the synthesis of proline as a by-product. We further show that PYCR1 activity is required for the successful maintenance of hypoxic regions by permitting continued TCA cycle activity, and that its loss leads to significantly increased hypoxia *in vivo* and in 3D culture, resulting in widespread cell death.

INTRODUCTION

Proline is a unique non-essential amino acid with critical roles in both protein structure and the cellular stress response (Phang et al., 2008). Although its function in the appropriate folding of polypeptides has long been understood, evidence over the past few years has suggested that it may play a central role in maintaining redox homeostasis in mammalian systems (Krishnan et al., 2008; Phang, 2019; Phang et al., 2008; Vettore et al., 2021).

Much of the balance of oxidizing and reducing potential within cells is achieved by two pyridine nucleotide couples: NAD^+:NADH and $NADP^+$:NADPH. The NAD^+:NADH redox couple are of particular importance in linking central carbon metabolism with ATP generation. One of the best defined cytosolic examples of this is the activity of lactate dehydrogenase (LDH), which balances the cytosolic NAD^+/NADH ratio through reduction of pyruvate (or oxidation of lactate) (Markert, 1984). However, the mitochondria are major contributors to the overall cellular NAD^+/NADH ratio, with the oxidizing activity of the tricarboxylic acid (TCA) cycle driving significant reduction of the mitochondrial NAD^+ pool. Under normal conditions, the high energy electrons from resulting NADH are used to generate the proton gradient across the inner mitochondrial membrane, which generates ATP through oxidative phosphorylation. However, NADH oxidation by the electron transport chain requires sufficient oxygen to act as the terminal electron acceptor. In conditions of low oxygen (hypoxia) the mitochondrial NADH/NAD^+ ratio increases, resulting in fragmentation of the TCA cycle and the need to shuttle reducing potential into the cytosol through the malate-aspartate shuttle (Frezza et al., 2011; LaNoue et al., 1973). The resulting shift in the cytosolic NAD^+/NADH ratio

This is an open access article under the CC BY license (http://creativecommons.org/licenses/by/4.0/).

results in an increase in lactate production (Eales et al., 2016). Importantly, hypoxia is a particularly prevalent factor in cancer growth, during which the rate of cell proliferation is often in excess of the delivery of oxygen via the dysfunctional tumor vasculature (Harris, 2002). In such conditions, the NAD^+/NADH ratio can become limiting for cell proliferation (Luengo et al., 2021), which may increase the activity of other redox-active pathways as a means of regenerating NAD^+.

The metabolism of proline is significantly linked to cellular redox state (Phang et al., 2008). Proline degradation is catalyzed by proline oxidase (also known as proline dehydrogenase [PRODH]), which reduces ubiquinone in the mitochondrial inner membrane via $FADH_2$ (Phang et al., 2010). This facilitates proton pumping and ATP synthesis, and can also generate reactive oxygen species through electron leakage. Synthesis of proline utilizes the reducing potential of either NADH or NADPH (De Ingeniis et al., 2012). This reaction is a two-step process, the first of these generating the intermediate glutamate 5-semialdehyde (GSA), which spontaneously cyclizes to form pyrroline 5-carboxylate (P5C). GSA can be formed either from glutamate by the action of P5C synthase (P5CS/ALDH18A1) coupled to the oxidation of NADPH, or from ornithine, which is coupled instead to the transamination of α-ketoglutarate (Burke et al., 2020). The second reaction is catalyzed by one of three isozymes with pyrroline 5-carboxylate reductase activity, known as PYCR1, PYCR2, and PYCR3/L. Importantly, while PYCR1 and PYCR2 are in the mitochondrial matrix, PYCR3 is known to be cytosolic, compartmentalizing aspects of this metabolic network (De Ingeniis et al., 2012). Previous studies have suggested that the activity of PRODH and PYCR enzymes might act together as a cycle, which could move proline, intermediates, and, therefore, redox equivalents between the mitochondria and the cytosol (Phang et al., 2012). However, it is likely that the activity of each of these enzymes is dependent on the cellular context and microenvironment. As an example, while metastatic cells have been shown to have an overall proline catabolic activity utilizing the energy generated via proline degradation to support the invasive process (Elia et al., 2017; Scott et al., 2019), fibroblasts stimulated with transforming growth factor β demonstrate increased proline synthesis (Schworer et al., 2020).

We and others recently suggested that proline biosynthesis through PYCR1 could be an alternative means by which mitochondria could continue to support TCA cycle oxidative activity while bypassing the need to pass the resulting electrons into the electron transport chain (Hollinshead et al., 2018; Schworer et al., 2020). There is some evidence that this could be true in conditions where oxygen is limiting due to reduction of the electron transport chain and increase in NADH/NAD^+ ratio in the mitochondrial matrix (Liu et al., 2020). In this study, we show that mitochondrial proline synthesis, specifically through PYCR1 activity, is essential to support hypoxic metabolism and viability. Loss of PYCR1 *in vivo* results in an unsustainable compensatory increase in respiration, cell death, and decreased tumor growth. We therefore propose that inhibition of PYCR1 is a viable target for killing cancer cells in hypoxic tumor regions, which are refractory to many conventional treatment approaches.

RESULTS

Hypoxia increases PYCR1-dependent proline synthesis and export from glutamine

Our previous research, and that of others, suggested that proline synthesis is increased in cells when cellular redox homeostasis is perturbed or when enhanced oxidation of mitochondrial NADH is required (Hollinshead et al., 2018; McGregor et al., 2020; Schworer et al., 2020). We previously hypothesized that this phenotype is likely to occur under conditions where the NADH/NAD^+ ratio becomes limiting for oxidizing TCA cycle activity, such as in hypoxia (Hollinshead et al., 2018). We therefore investigated whether this was the case by incubating human cells in either normoxia (21% O_2) or increasing severity of hypoxia (1% and 0.3% O_2). We found that in a number of cell models representing triple-negative breast cancer (SUM159PT, HCC1806), medulloblastoma (ONS), and bone marrow stromal cells (HS-5), hypoxia enhanced proline synthesis and efflux into the medium (Figures 1A and S1A). Importantly, this appeared to occur without a consistent change in expression of either of the key biosynthetic enzymes, PYCR1 or PYCR2 (Figures 1B, 1C, and S1B), suggesting that the normoxic expression of these enzymes is capable of supporting flux through the pathway in excess of that observed under normoxic conditions.

We previously showed that PYCR1 is responsible for enhanced proline biosynthesis from glutamine in cells expressing the oncogenic IDH1 R132H mutation (Hollinshead et al., 2018). We therefore first examined whether glutamine-derived proline synthesis also underpinned the hypoxia-driven response. SUM159PT cells were incubated with [U-^{13}C]glutamine in normoxia or hypoxia (1% and 0.3% O_2) and demonstrated the expected hypoxia-induced reductions in glutamine oxidation (M + 4 isotopomer of aspartate and citrate; Figures S1C and S1D) as well as a relative increase in the contribution of reductive carboxylation of glutamine to the citrate pool (M + 5 isotopomer; Figure S1D). Under the same conditions, we noted that glutamine-derived proline exported into the medium increased significantly with increasing severity of hypoxia (Figure 1D), indicative of increased mitochondrial synthesis. We consider the ratio of fully labeled glutamate to proline a surrogate of the overall activity of this pathway (Figure 1B). Although this ratio was significantly increased in 1% O_2, it returned to normoxic levels in 0.3% O_2 (Figure 1E). Given that export of proline into the medium was significantly enhanced in these conditions (Figure 1D), we concluded that this likely reflected a further adaptation to increased proline synthesis under severe hypoxia to relieve the product inhibition on PYCR enzymes that may otherwise be suboptimal for continued cell viability (De Ingeniis et al., 2012).

Two constitutively expressed mitochondrial isozymes, PYCR1 and PYCR2, are responsible for the second step in the synthesis of proline from glutamate (Figure 1B). To elucidate which of these might demonstrate a hypoxia-mediated induction of activity, we knocked down PYCR1 or PYCR2 (Figure S1E) and examined the synthesis of proline in normoxia and hypoxia. We observed that knockdown of PYCR1 resulted in a significant decrease of intracellular proline in normoxia and an inability to respond to hypoxia (Figure 1F). Conversely, knockdown of PYCR2 had little effect (Figure 1F). In light of our previous data suggesting that PYCR1

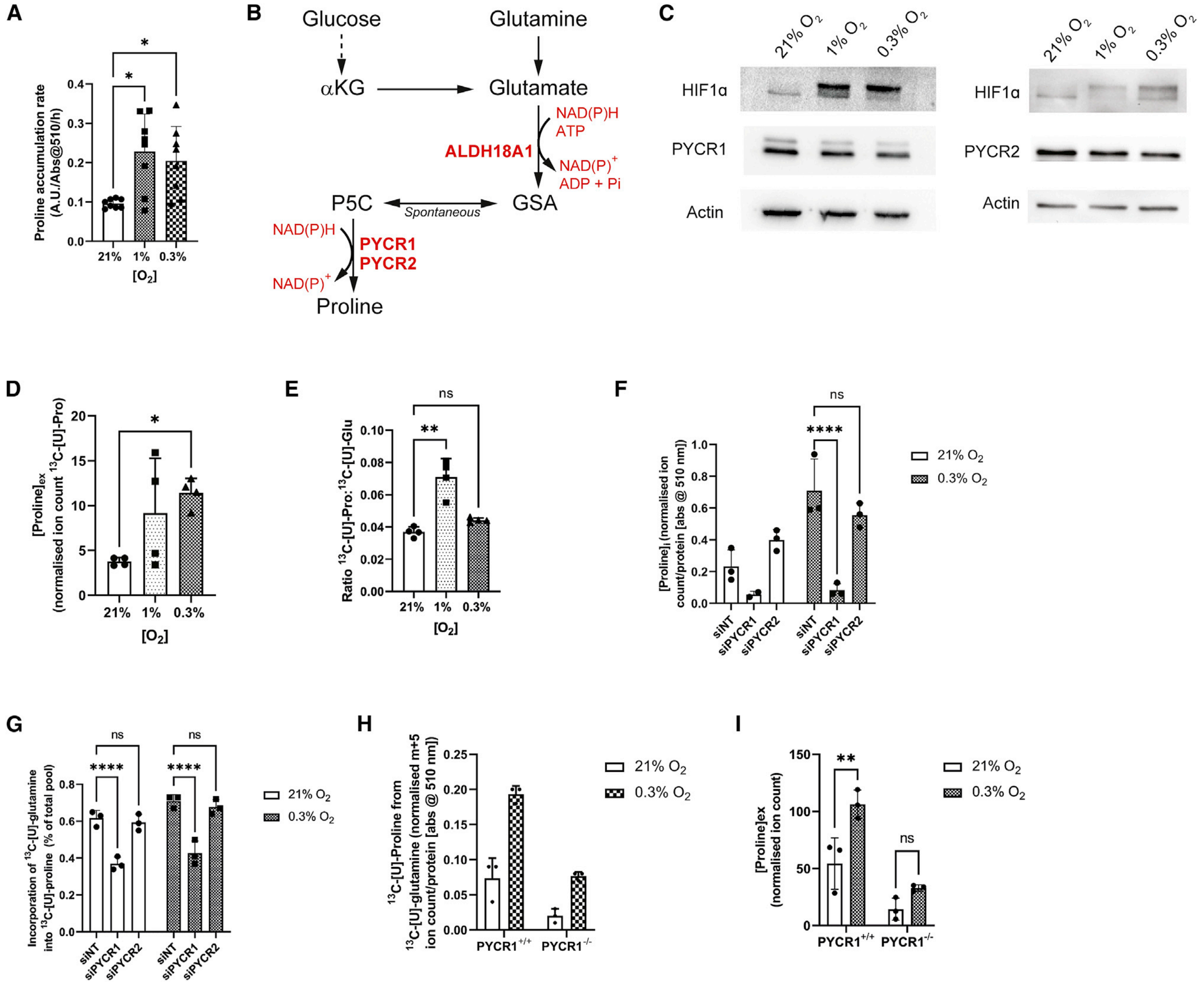

Figure 1. Hypoxia increases PYCR1-dependent proline synthesis and export from glutamine

(A) Intracellular proline abundance in SUM159PT cells is increased with hypoxia (n = 8, presented as mean ± SD). *, $p < 0.05$

(B) Diagram of proline synthesis from glucose and glutamine. αKG, α-ketoglutarate; ALDH18A1, aldehyde dehydrogenase 18 family member A1 (pyrroline-5-carboxylate synthase [P5CS]); PYCR1, pyrroline-5-carboxylate reductase 1; PYCR2, pyrroline-5-carboxylate reductase 2.

(C) Protein expression of proline synthetic enzymes does not change with decreasing oxygen tension in SUM159PT cells.

(D) Extracellular proline abundance is significantly increased in 0.3% O_2 in SUM15PT cells (n = 4, presented as mean ± SD). *, $p < 0.05$

(E) The ratio of [U-^{13}C]proline to [U-^{13}C]glutamate from [U-^{13}C]glutamine tracing is significantly increased in 1% O_2 (n = 4, presented as mean ± SD). **, $p < 0.01$

(F) Extracellular proline concentration in SUM159PT cells transfected with siPYCR1 is significantly decreased in hypoxia (0.3%) compared with siNT. siPYCR2 does not significantly alter the proline concentration (n = 3, presented as mean ± SD). ****, $p < 0.0001$

(G) The contribution of glutamine to the total proline pool (%) is significantly reduced with siPYCR1 and not with siPYCR2 (n = 3, presented as mean ± SD). ****, $p < 0.0001$

(H) In hypoxia (0.3% O_2), abundance of [U-^{13}C]proline from [U-^{13}C]glutamine is increased in SUM159PT PYCR1$^{+/+}$ cells. This increase is not seen in PYCR1$^{-/-}$ cells (n = 3, presented as mean ± SD).

(I) Total extracellular proline is increased in hypoxia (0.3%) in PYCR1$^{+/+}$ cells. This increase is not seen in PYCR1$^{-/-}$ cells (n = 3, presented as mean ± SD). **, $p < 0.01$. αKG, α-ketoglutarate; ALDH18A1, aldehyde dehydrogenase 18 family member A1; GSA, glutamate semialdehyde; P5C, pyrroline 5-carboxylate; PYCR1, P5C reductase 1; PYCR2, P5C reductase 2.

was the major determinant of IDH1^{R132H}-driven increased proline synthesis from glutamine in glioma (Hollinshead et al., 2018), we also assessed glutamine-derived proline and noted that knockdown of PYCR1 but not PYCR2 led to a decrease in the proportion of proline derived from glutamine in both normoxia and hypoxia (Figure 1G). To extend these findings to an alternative cell model, we utilized a previously described CRISPR-engineered *PYCR1*$^{-/-}$ SUM159PT cell line (Figure S1F) with $^{13}C_5$-glutamine (Loayza-Puch et al., 2016). In the absence of PYCR1, intracellular proline synthesis from glutamine was

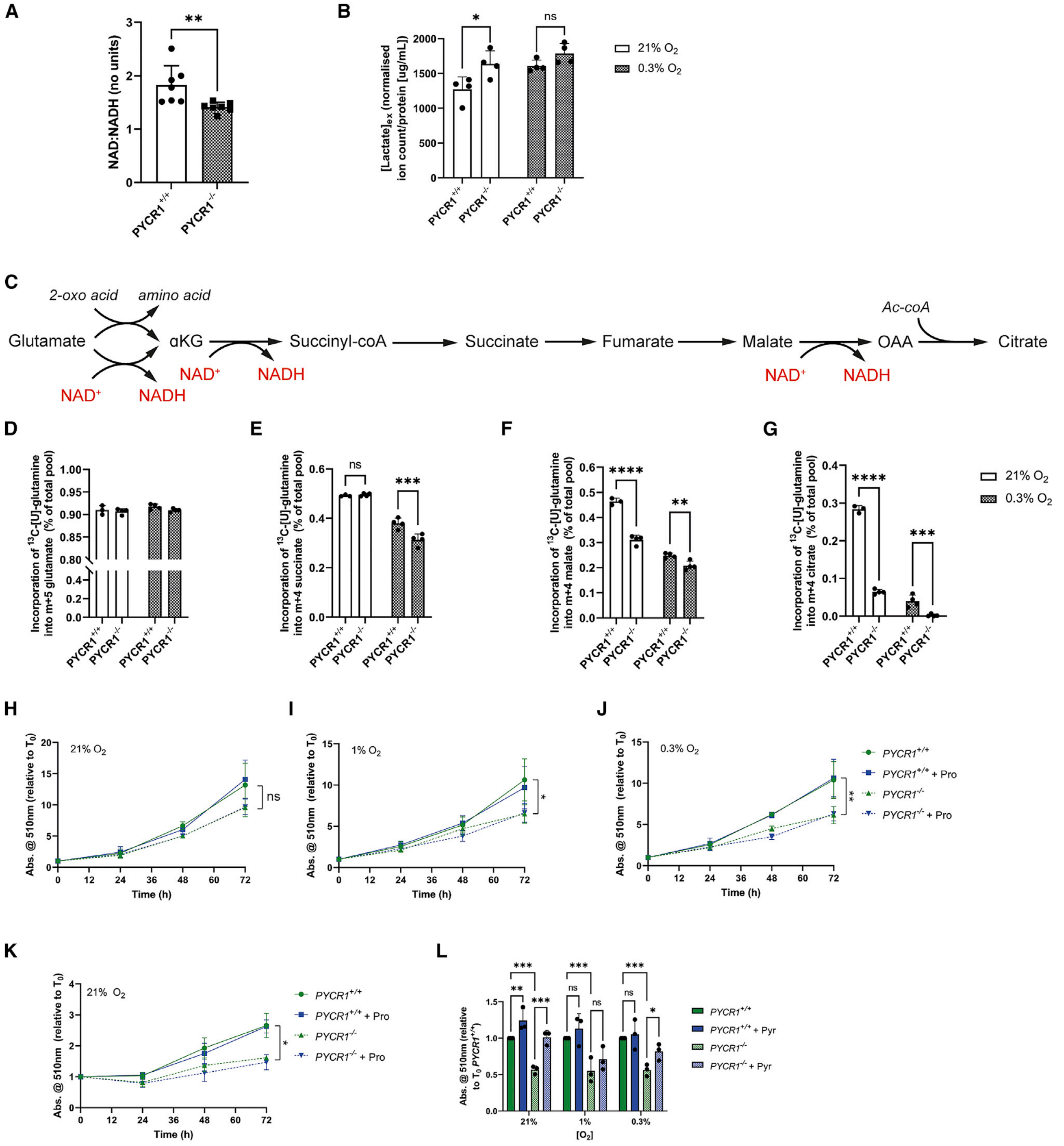

Figure 2. PYCR1 activity supports normal TCA cycle activity and proliferation

(A) The NAD/NADH ratio is significantly reduced in PYCR1$^{-/-}$ cells (n = 7, presented as mean ± SD). **, $p < 0.01$

(B) Total extracellular lactate (ion count/protein) is increased in PYCR1$^{-/-}$ cells compared with PYCR1$^{+/+}$. In hypoxia (0.3%) there is no significant change (n = 4, presented as mean ± SD). *, $p < 0.05$

(C) Diagram showing glutamate entry into the TCA cycle, highlighting NAD-requiring steps.

(D) Percentage incorporation of [U-^{13}C]glutamine into M + 5 glutamate is unchanged between conditions (n = 4, presented as mean ± SD).

(E) Percentage incorporation of [U-^{13}C]glutamine into M + 4 succinate is decreased in the PYCR1$^{-/-}$ cells in hypoxia (n = 4, presented as mean ± SD). ***, $p < 0.001$

(F) Percentage incorporation of [U-^{13}C]glutamine into M + 4 malate is reduced in both oxygen tensions in the PYCR1$^{-/-}$ cells (n = 4, presented as mean ± SD). **, $p < 0.01$; ****, $p < 0.0001$

(legend continued on next page)

significantly reduced but, importantly, the response to hypoxia was also blunted (Figures 1H and S1G). This was also observed in the medium, in which loss of PYCR1 ameliorated the hypoxia-induced efflux of proline (Figure 1I). Our data therefore shows that hypoxia leads to an increase in proline synthesis and export, which is mainly derived from glutamine and is dependent on PYCR1.

PYCR1 activity supports normal TCA cycle activity and proliferation

We and others have suggested that mitochondrial proline synthesis may be an important mechanism to buffer changes in the mitochondrial NAD^+/NADH ratio (Hollinshead et al., 2018; Schworer et al., 2020). We therefore investigated whether loss of PYCR1 led to a change in the NAD^+/NADH ratio in this model, and found that this was indeed the case (Figure 2A), supporting a role for PYCR1 as part of an obligate mitochondrial NAD^+ regeneration system. We also tested whether loss of PYCR1 activity might affect the $NADP^+$/NADPH ratio, as these enzymes are also capable of oxidizing this pyridine nucleotide. To this end, we measured the reduced/oxidized glutathione (GSH/GSSG) ratio—a surrogate of the $NADP^+$/NADPH ratio due to the tight coupling of these systems—and found that it was unchanged in either the small interfering RNA or knockout cell model (Figures S2A and S2B). Due to the specific effect of loss of PYCR1 activity on the NAD^+/NADH ratio, we hypothesized that this may elicit a compensatory increase in respiration or perturbation of the malate-aspartate shuttle, the latter of which could be observed by increased lactate production and export due to enhanced cytosolic NAD^+ regeneration. We found that while respiration was not significantly affected (Figures S2C and S2D), lactate synthesis and efflux was increased in cells deficient for PYCR1 activity in normoxia (Figure 2B). Due to this incomplete compensation, we further examined whether the loss of PYCR1-mediated NADH-oxidizing activity may reduce oxidative TCA cycle flux (Figure 2C). We incubated cells with [U-^{13}C]glutamine and observed reduced incorporation into TCA cycle metabolites in hypoxia after the first NADH-generating steps (synthesis of succinate; Figures 2D, 2E, S2E, and S2F), which was further decreased in subsequent steps (Figures 2F, 2G, S2G, and S2H). Of note, the synthesis of citrate from glutamine, which is downstream of a further NAD^+-dependent step (malate dehydrogenase), was reduced in normoxia and almost entirely absent in hypoxia (Figures 2G and S2H). The reduction in TCA cycle activity after loss of PYCR1 activity could be expected to reduce cell proliferation (Loayza-Puch et al., 2016), as TCA cycle activity (and the synthesis of aspartate) is critical for cellular anabolism (Birsoy et al., 2015; Sullivan et al., 2015). We monitored proliferation in PYCR1$^{-/-}$ cells and noted reduced proliferation in both normoxia and hypoxia (Figures 2H–2J), consistent with other recent data (Milne et al., 2019). As reduced proliferation could be a result of either perturbed redox homeostasis or deficiency in intracellular proline (Sahu et al., 2016), we tested both of these hypotheses. First, we cultured the cells in the presence of exogenous proline and found that despite efficient uptake (Figure S2I), the proliferative defect remained (Figures 2H–2J). However, the fact that the PYCR1$^{-/-}$ cells remained capable of proliferation suggested that the proline normally present in the medium (150 μM) and/or the activities of PYCR2/3 were sufficient to support proliferation. To test this, we cultured cells in an alternative medium without proline and found that the relative proliferative defect between PYCR1$^{+/+}$ and PYCR1$^{-/-}$ cells was still observed, and the phenotype was still not rescued with additional proline (Figure 2K). These data therefore suggested that the remaining PYCR enzymes were capable of synthesizing sufficient proline to support this reduced proliferative rate. To test whether further metabolic support of cellular redox homeostasis might rescue the proliferative defect, we cultured the cells in medium containing additional exogenous pyruvate, which could support increased rates of NADH oxidation. We found that in contrast to proline supplementation, exogenous pyruvate did at least partially rescue proliferation in both normoxia and hypoxia (Figure 2L). Overall, these data strongly support the hypothesis that PYCR1 supports proliferation through maintaining efficient oxidative TCA cycle activity, but in conditions where redox cannot be buffered, such as when oxygen is diffusion limited, PYCR1 may become essential.

Loss of PYCR1 in 3D culture leads to redox imbalance and excessive oxygen consumption

To more accurately recapitulate the multiple metabolite diffusion gradients present *in vivo*, most importantly that of oxygen, we extended our investigation to a three-dimensional (3D) spheroid model. When these were grown using the soft-agar approach, we found that loss of PYCR1 led to a substantial reduction in spheroid size (Figures 3A and 3B). However, due to the duration of this experiment (10 days), the previously described effect on cell proliferation (Figures 2H–2J; Loayza-Puch et al., 2016; Sahu et al., 2016) made it difficult to appropriately interpret the results. We therefore used an alternative method to generate the spheroids, which allows them to rapidly form from a pre-plated number of cells, rather than proliferating to reach the desired cell number (Ivascu and Kubbies, 2006). While spheroids formed efficiently, they remained significantly smaller (Figures 3C and 3D). Importantly, the spheroids did reach a size at which oxygen diffusion was limiting, resulting in hypoxia (pimonidazole staining;

(G) Percentage incorporation of [U-^{13}C]glutamine into M + 4 citrate is reduced in both oxygen tensions in the PYCR1$^{-/-}$ cells and is almost undetectable in 0.3% oxygen (n = 4, presented as mean ± SD). ***, $p < 0.001$; ****, $p < 0.0001$

(H–J) Cell growth with PYCR1 loss. Statistical analysis shown compares PYCR1$^{+/+}$ and PYCR1$^{-/-}$ cells without proline. Comparison of growth of either cell type with or without proline was not significant in 21% O_2 (H), $p < 0.05$ in 1% O_2 (I), and $p < 0.01$ in 0.3% O_2 (J) in DMEM/Nutrient Mixture F-12 Ham. Growth was not altered by the addition of 2 mM exogenous proline in any oxygen tension (n = 3, presented as mean ± SEM).

(K) Cell growth of PYCR1$^{-/-}$ cells is maintained in DMEM flux medium without proline and is not rescued with the addition of exogenous proline (n = 3, presented as mean ± SEM). Statistical analysis shown compares PYCR1$^{+/+}$ and PYCR1$^{-/-}$ cells without proline. *, $p < 0.05$. Comparison of growth of either cell type with or without proline was not significant.

(L) Cell growth of PYCR1$^{-/-}$ cells can be rescued with the addition of 2 mM sodium pyruvate (n = 3, presented as mean ± SD).*, $p < 0.05$; **, $p < 0.01$; ***, $p < 0.0001$

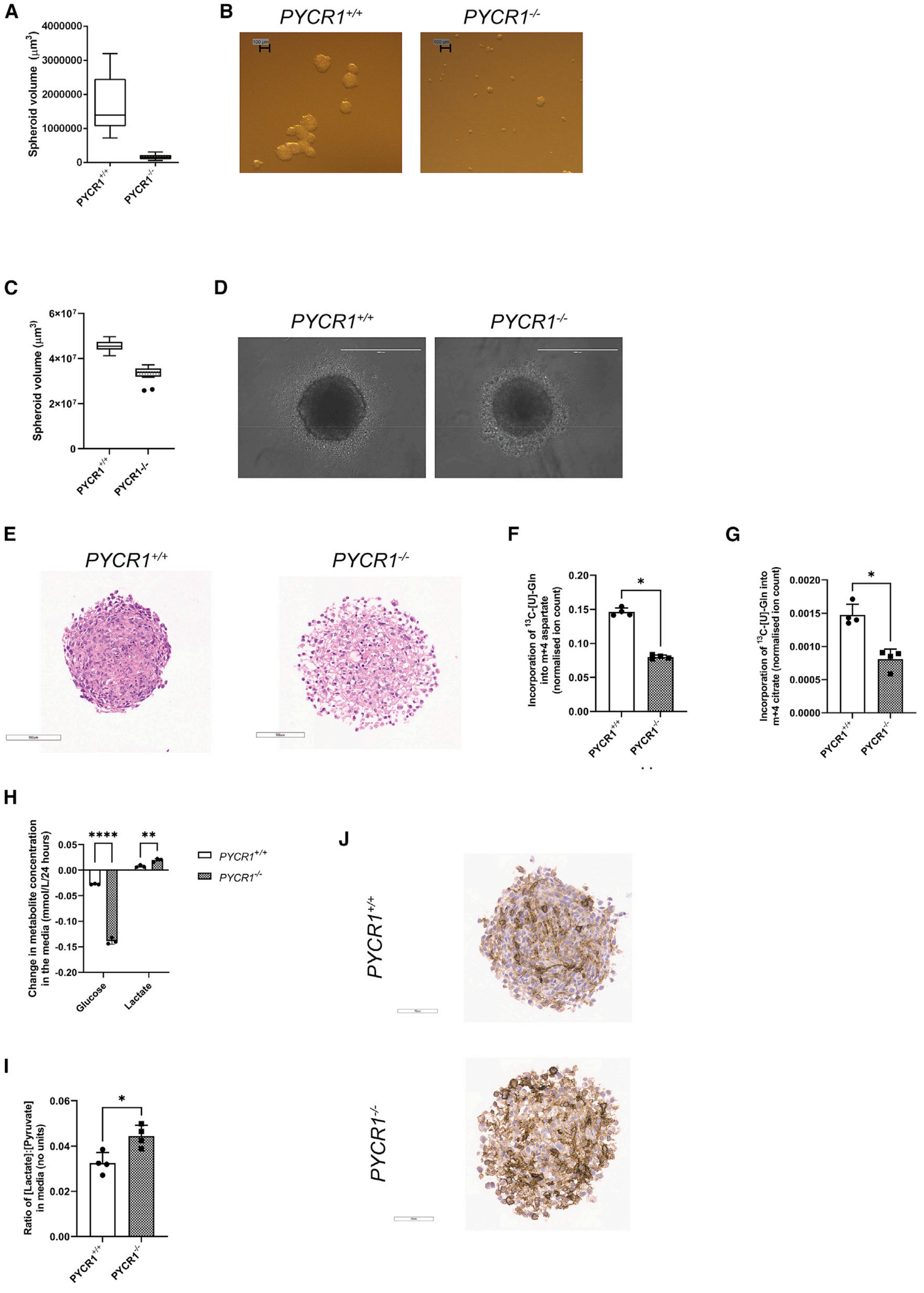

Figure 3. Loss of PYCR1 in 3D culture leads to redox imbalance and excessive oxygen consumption

(A) Volume of PYCR1$^{-/-}$ and PYCR1$^{+/+}$ spheroids grown on agarose-coated plates (n = 3, presented with Tukey whiskers).

(B) Representative images of spheroids from (A). Scale bars, 100 μm.

(legend continued on next page)

Figure S3A) and increased expression of the hypoxia-inducible enzymes GLUT1 and carbonic anhydrase 9 (CA9; Figures S3B and 3J). We performed further histological analysis of similarly sized spheroids and found that the PYCR1$^{-/-}$ spheroids had an altered morphology, characterized by a less compact center containing significant numbers of clear inclusions (Figure 3E). We first investigated whether loss of PYCR1 in spheroids also led to decreased proline synthesis and export, as observed in two-dimensional (2D) culture. We incubated spheroids with [U-^{13}C]glutamine and found that the incorporation of glutamine into proline was decreased both in the spheroids and the medium (Figures S3C and S3D). In light of this, we also tested whether exogenous proline could rescue spheroid growth but, in line with our 2D results, there was little effect (Figure S3E). We subsequently investigated the metabolic activity of PYCR1-deficient spheroids and found that, consistent with the previously observed defect in TCA cycle oxidative activity, PYCR1-deficient spheroids contained less glutamine-derived aspartate and citrate (Figures 3F and 3G). We therefore also investigated whether glycolysis may be increased as a result of the mitochondrial dysfunction in response to loss of PYCR1 and found that glucose consumption and lactate export increased (Figure 3H). Finally, as we showed in 2D that loss of PYCR1 activity altered the NAD$^+$/NADH ratio, we examined the lactate/pyruvate ratio, a well-described surrogate for the cytosolic NAD$^+$/NADH ratio, in the medium. Consistent with the reduced oxidative TCA cycle activity, we noted an increase in the ratio in the medium from spheroids, indicative of an increased need of PYCR1$^{-/-}$ cells to regenerate NAD$^+$ in the cytosol (Figure 3I). Given the significant changes in oxidative metabolism observed, we hypothesized that mitochondrial respiration may be increased as an attempt to compensate, leading to a steeper oxygen gradient across the spheroid and greater area of hypoxia. We therefore stained the spheroids for CA9, a well-characterized hypoxia-responsive protein (Wykoff et al., 2001). While CA9 was present in the wild-type spheroids as expected, levels were significantly higher and in greater cell numbers when PYCR1 was absent (Figure 3J). These results suggested that PYCR1 is a critical component of the hypoxic response in the metabolic network and that its loss leads to widespread dysfunction.

PYCR1 is essential for maintenance of viable hypoxic regions and tumor growth

To investigate the role of PYCR1 in the hypoxic regions of tumors, we engineered a cell model in which PYCR1 could be inducibly knocked down, thereby minimizing the effect of PYCR1 on other cell processes during tumor initiation and initial growth. We found that in our model, PYCR1 expression could be rapidly reduced after induction of the short hairpin RNA construct with doxycycline (DOX), with no effect on the control cell model either *in vitro* or *in vivo* (shNT; Figures S4A–S4D). Using this cell model, we first investigated the effect of short-term PYCR1 knockdown on hypoxia and hypoxic signaling in tumors after a single dose of DOX. We found that the extent of hypoxia increased significantly across the tumor in response to knockdown of PYCR1 (Figures 4A and 4E). This was accompanied by significant changes in hypoxia signaling, indicated by increased expression of the HIF1 target genes, CA9 and GLUT1 (Figures 4B, 4C, 4F, 4G, and S4E). Finally, we assessed the effect of enhanced hypoxic signaling on tumor angiogenesis (measured by CD31-positive cells) and observed a surprising relative decrease in CD31-positive cells, suggesting reduced angiogenic signaling (Figures 4D and 4H). However, this latter marker was not accompanied by a reduction in apparent blood flow (Figure S4F), suggesting that this time point may be insufficient to elicit an angiogenic response of magnitude sufficient to reduce perfusion. As we had observed a significant increase in tumoral hypoxia over a relatively short time frame, we investigated whether this may affect tumor phenotype. We found again that a single dose of DOX resulted in reduced tumor cell proliferation (Ki67 staining; Figures 5A and 5D) only in the shPYCR1 tumors, which also demonstrated increased apoptotic cell death (cleaved caspase-3; Figures 5B, 5E, and S5A). These significant changes in cell phenotype were mirrored by extensive areas of histological necrosis indicative of substantial cell death (Figures 5C and 5F). Given these data, we examined the effect of chronic dosing of mice with DOX to provide long-term knockdown of PYCR1 on established tumor growth. We found that, in agreement with the acute dosing study, only the shPYCR1 + DOX-treated tumors showed a significant change in growth, increasing the time taken to reach an average of 500 mm^3 by over 30% (Figures 5G, 5H, and S5B–S5I).

Overall, our studies show that PYCR1 supports normal mitochondrial metabolism by contributing to redox homeostasis. In hypoxia, PYCR1 becomes an essential oxygen-sparing enzyme, permitting continued TCA cycle activity and reducing lactate production. Loss of PYCR1 in cells results in significant metabolic rewiring, and loss of PYCR1 in tumors results in cell death and reduced tumor growth.

DISCUSSION

There is increasing evidence supporting the hypothesis that activity of the proline synthesis and degradation pathways is

(C) Volume of PYCR1$^{-/-}$ and PYCR1$^{+/+}$ spheroids at 72 h grown in U-bottomed plates (n = 3, presented with Tukey whiskers).

(D) Representative images of spheroids from (C). Scale bars, 400 μm.

(E) Hematoxylin and eosin (H&E) staining of sectioned spheroids showing that PYCR1$^{-/-}$ spheroids have an altered morphology, with less densely packed cells and more rounded nuclei. Scale bars, 100 μm.

(F) The percentage incorporation of [U-^{13}C]glutamine into M + 4 aspartate is lower in PYCR1$^{-/-}$ spheroids, presented as mean ± SD. *, $p < 0.05$

(G) The percentage incorporation of [U-^{13}C]glutamine into M + 4 citrate is lower in PYCR1$^{-/-}$ spheroids (n = 4 [× 24 spheroids], presented as mean ± SD). *, $p < 0.05$

(H) Glucose consumption is increased in the PYCR1$^{-/-}$ spheroids while lactate export into the medium is increased (n = 3 [× 24 spheroids], presented as mean ± SD). **, $p < 0.01$; ****, $p < 0.0001$

(I) The ratio extracellular lactate to extracellular pyruvate is higher in PYCR1$^{-/-}$ spheroids (n = 4 [× 24 spheroids], presented as mean ± SD). *, $p < 0.05$

(J) PYCR1$^{+/+}$ and PYCR1$^{-/-}$ spheroids stained for carbonic anhydrase IX expression, as a marker of hypoxic cells. More intense and diffuse staining is seen in the PYCR1$^{-/-}$ spheroids. Scale bars, 70 μm.

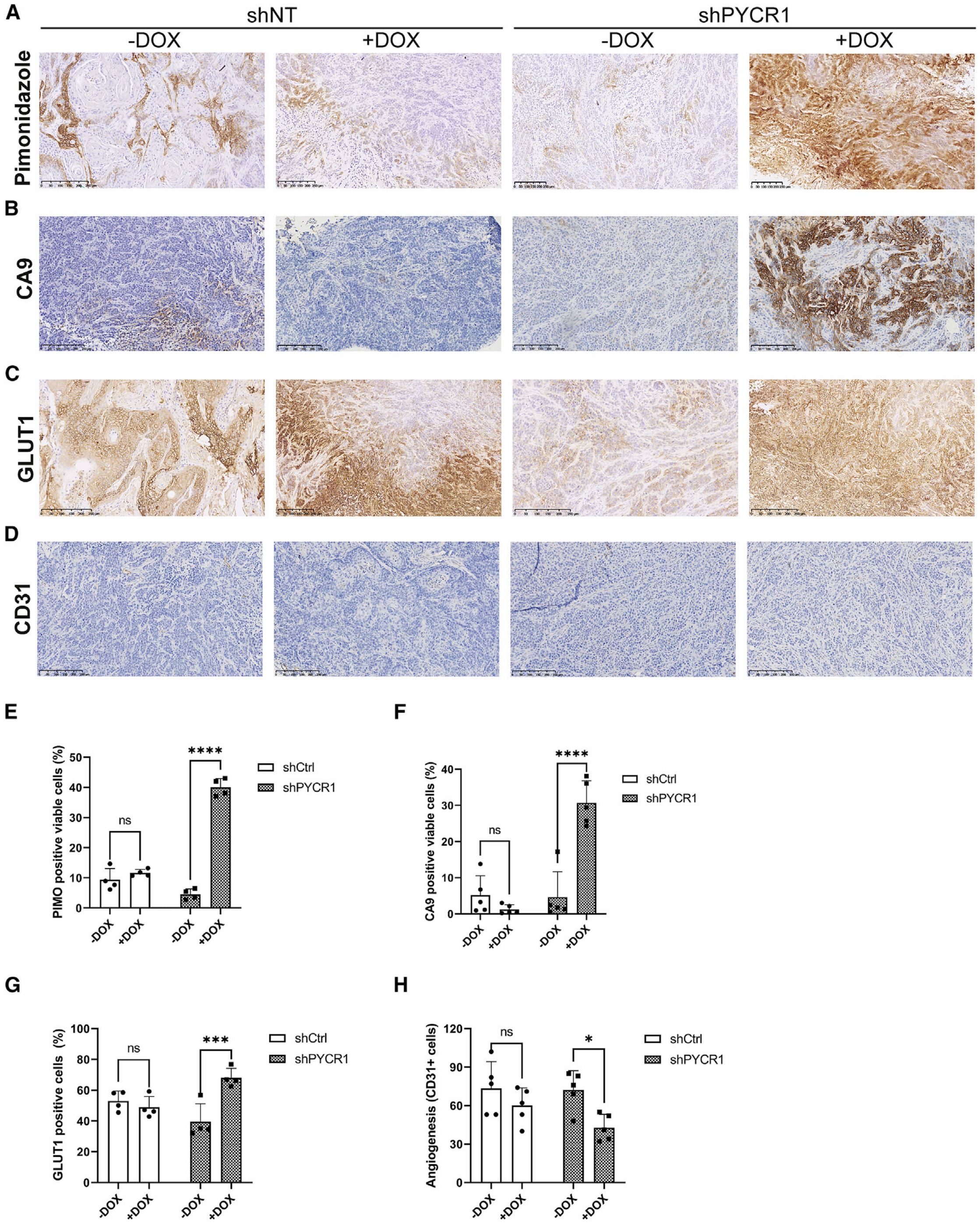

Figure 4. PYCR1 loss leads to increased tumor hypoxia

(A) Representative images showing pimonidazole staining in xenografts.

(B) Representative images showing CA9 staining in xenografts.

(C) Representative images showing GLUT1 staining in xenografts.

(D) Representative images showing CD31 staining in xenografts.

(E) Quantification of pimonidazole staining as percentage of positive cells, presented as mean ± SD. ****, $p < 0.0001$. Doxycycline (DOX)-induced shPYCR1 xenografts have significantly higher staining positivity, indicating that PYCR1 loss increases hypoxia.

(F) Quantification of CA9 staining as percentage of positive cells, presented as mean ± SD. ****, $p < 0.0001$. . Doxycycline-induced shPYCR1 xenografts have significantly higher staining positivity.

(legend continued on next page)

important in certain cellular and environmental conditions to modulate the redox state in mammalian cells. PYCR1 activity in particular has been shown to be responsive to the mitochondrial NAD/NADH ratio (Hollinshead et al., 2018; Schworer et al., 2020).

Our data suggest that PYCR1 represents a critical component of an obligate mitochondrial NADH-oxidizing pathway, which under limiting oxygen conditions becomes essential. We also show that despite being expressed constitutively alongside PYCR2, these enzymes are non-redundant (Figures 1F and 1G). Although both of these isozymes catalyze the same reaction and are localized to the mitochondria, it is thought that PYCR2 has a higher affinity for NADPH as a co-factor (De Ingeniis et al., 2012). However, more work is needed to understand the regulation and distinct roles of these two isozymes. It should also be noted that the synthesis of proline through PYCR1 is still dependent on the availability of NADP for reduction of glutamate, as cells lacking NADK2 have sufficiently impaired proline synthesis to become proline auxotrophic (Tran et al., 2021; Zhu et al., 2021). Importantly, expression of PYCR1 was not increased in hypoxia (Figure 1C). This suggests that PYCR1 is constitutively expressed at a level that allows it to respond very rapidly to acute changes in mitochondrial redox homeostasis, providing the ability to maintain a functional mitochondrial network in the short term while a longer-term transcriptional framework is put in place through hypoxia-activated signaling mechanisms (such as hypoxia-inducible factor 1).

Proline synthesized through the reduction of glutamate appears in our models to be in excess of cellular anabolic requirements and is exported into the medium, much like the reduction of pyruvate to lactate in the cytosol. PYCR1 activity therefore appears to functionally uncouple the TCA cycle from ATP synthesis, a concept previously suggested to be permissive to enhanced proliferation (Luengo et al., 2021) and which our data support. In line with this, we find that PYCR1$^{-/-}$ cells produce increased extracellular lactate in normoxia, suggesting that PYCR1 supports the normal function of mitochondrial redox shuttles such as the malate-aspartate shuttle, which is also important for cellular anabolism. However, our data also suggest that under conditions of severe hypoxia, further mechanisms to maintain PYCR1 activity by avoiding previously described product inhibition may be in place (De Ingeniis et al., 2012), which could include the upregulation of an as yet unidentified mitochondrial transporter for this amino acid (Figures 1D and 1E).

In 3D culture, which is more representative of the oxygen and nutrient gradients observed in the tumor microenvironment, we also found that PYCR1 loss reduced proline export. The knockout also affected spheroid morphology, increased hypoxia, and resulted in a metabolic phenotype indicative of significant metabolic dysfunction. Finally, we showed *in vivo* that loss of PYCR1 significantly decreased growth in a mouse xenograft model. We found that proliferation was reduced while hypoxia and cell death were increased. This further suggests that proline synthesis through PYCR1 is conditionally essential when cellular redox state is reduced, such as in a hypoxic microenvironment.

In summary, in parallel with the original hypothesis supporting the use of anti-angiogenic drugs in tumors, our data suggest that inhibition of PYCR1 may push hypoxic tumor regions into a non-viable state, making the overall tumor more amenable to conventional therapies whose efficacy suffers in hypoxia. However, further work is needed to define the roles of other PYCR isozymes in both normoxia and hypoxia, as our data and that of others clearly show that the metabolic network surrounding proline is complex and remains poorly understood.

Limitations of the study

While we showed much of the downstream biochemical effects of reduced PYCR1 activity in normoxia and hypoxia *in vitro*, we were only able to show some of these markers in the subsequent *in vivo* study due to the cross-species, multicellular nature of the orthotopic xenograft model used. The role of PYCR1 specifically in redox homeostasis could therefore not be confirmed *in vivo*. In terms of translation of these findings, the implications of pharmacological inhibition of PYCR1 are not yet entirely clear, as studies did not address this. Although PYCR1 deficiency led to a necrotic phenotype, it also increased tumoral hypoxia that itself could increase hypoxia-induced phenotypes such as therapy resistance. Further studies are therefore needed to clarify how PYCR1 inhibition could contribute to future therapeutic interventions.

STAR★METHODS

Detailed methods are provided in the online version of this paper and include the following:

- KEY RESOURCES TABLE
- RESOURCE AVAILABILITY
  - Lead contact
  - Materials availability
  - Data and code availability
- EXPERIMENTAL MODEL AND SUBJECT DETAILS
  - Cell culture
  - *In vivo* model
- METHOD DETAILS
  - Spheroids
  - Metabolic tracing
  - Glucose and lactate consumption
  - Bicinchoninic acid (BCA) protein assay
  - Derivatization and GCMS
  - Western blotting
  - Cell number and GCMS data normalization values
  - Oxygen consumption
  - GSH:GSSG assay
  - NAD^+:NADH assay
  - Immunohistochemistry
- QUANTIFICATION AND STATISTICAL ANALYSIS

(G) Quantification of GLUT1 staining, as percentage of positive cell, presented as mean ± SD. ***, $p < 0.001$. s. Doxycycline-induced shPYCR1 xenografts have significantly higher staining positivity.

(H) Quantification of CD31 staining, presented as mean ± SD. *, $p < 0.05$. . Doxycycline-induced shPYCR1 xenografts show a small but statistically significant decrease in staining positivity, suggesting that angiogenesis is impaired by PYCR1 loss.

All scale bars represent 250 µm. All experiments: n = 5.

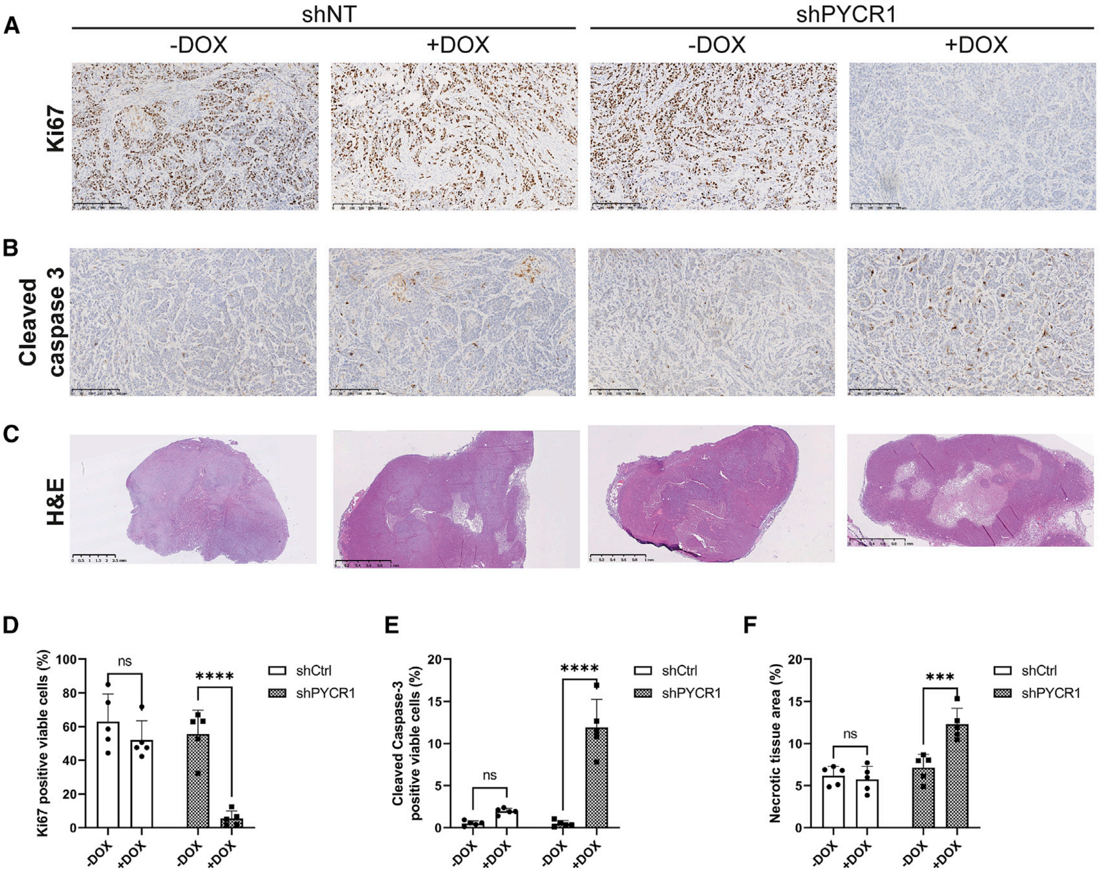

G

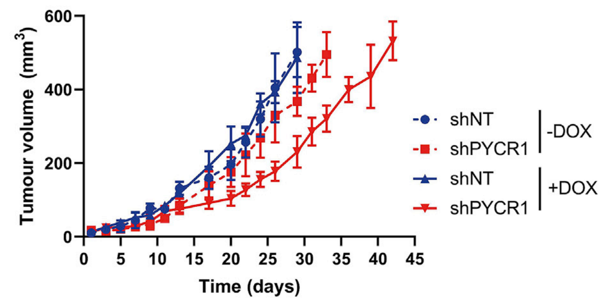

H

Growth Curve Comparison	*p* value	*t* value
shNT / shNT + DOX	0.37	0.97
shNT / shPYCR1	0.24	1.3
shNT / shPYCR1 + DOX	0.006	5.77
shNT + DOX / shPYCR1	0.64	0.47
shNT + DOX / shPYCR1 + DOX	0.001	6.75
shPYCR1 / shPYCR1 + DOX	0.006	5.32

Figure 5. PYCR1 is essential for maintenance of viable hypoxic tumor regions

(A–C) Representative images showing (A) Ki67, (B) cleaved caspase-3, and (C) H&E staining in xenografts (n = 5 for all conditions).
(D) Quantification of Ki67 positive cells as percentage of viable cells, presented as mean ± SD. ****, $p < 0.0001$.
(E) Quantification of cleaved caspase-3 positive cells as percentage of viable cells, presented as mean ± SD. ****, $p < 0.0001$. Doxycycline-induced shPYCR1 xenografts have significantly higher staining positivity, indicating that PYCR1 loss increases apoptosis.
(F) Quantification of necrotic area as percentage of total xenograft tumor area, presented as mean ± SD. ***, $p < 0.001$. Doxcycline-induced shPYCR1 xenografts have significantly higher percentage necrotic area.
(G) Tumor volume over time; shows doxycycline-induced shPYCR1 xenografts take more days to reach endpoint (500mm3) (n = 7, presented as mean ± SD).
(H) Significance values for tumor growth curve. p values reach significance for doxycycline-induced shPYCR1 comparison with shNT, doxycycline-induced shNT and shPYCR1. Scale bar = 250 μM (A), 1 mm (C; shPYCR1 + DOX) or 2.5 mm (C; shNT ±DOX & shPYCR1 – DOX).

SUPPLEMENTAL INFORMATION

Supplemental information can be found online at https://doi.org/10.1016/j.celrep.2022.110320.

ACKNOWLEDGMENTS

The authors would like to acknowledge funding from Cancer Research UK to R.L.W., C.E.G., K.L.E., E.V.-S., L.A.V., and P.D.W. (C42109/A26982 and

CellPress
OPEN ACCESS

C42109/A24747). We would also like to acknowledge Professor Reuven Agami, who kindly gifted the PYCR1$^{-/-}$ line and control. The graphical abstract was created with BioRender.com. Finally, we would like to acknowledge the support and resources of the Birmingham Metabolic Tracer Analysis Core.

AUTHOR CONTRIBUTIONS

R.L.W., E.B., A.S., H.L., A.L.H., and D.A.T. conceptualized the study and designed the methodology. R.L.W., E.B., C.E.G., J.R., H.R., K.L.E., K.-L.E., H.V., E.V.-S., L.A.V., P.D.W., F.C., and C.N. performed investigations. E.V.-S., E.B., R.L.W., K.-L.E., H.V., C.N., and D.A.T. performed formal analysis and data visualization. G.G.L., D.J.H., A.S., H.L., C.N., A.L.H., and D.A.T. provided resources. R.L.W., E.B., J.B., C.N., and D.A.T. wrote the original manuscript draft, while all authors contributed to reviewing and editing of the manuscript.

DECLARATION OF INTERESTS

The authors declare no competing interests.

Received: July 2, 2021
Revised: November 12, 2021
Accepted: January 10, 2022
Published: February 1, 2022

REFERENCES

Birsoy, K., Wang, T., Chen, W.W., Freinkman, E., Abu-Remaileh, M., and Sabatini, D.M. (2015). An essential role of the mitochondrial electron transport chain in cell proliferation is to enable aspartate synthesis. Cell *162*, 540–551.

Burke, L., Guterman, I., Palacios Gallego, R., Britton, R.G., Burschowsky, D., Tufarelli, C., and Rufini, A. (2020). The Janus-like role of proline metabolism in cancer. Cell Death Discov. *6*, 104.

De Ingeniis, J., Ratnikov, B., Richardson, A.D., Scott, D.A., Aza-Blanc, P., De, S.K., Kazanov, M., Pellecchia, M., Ronai, Z., Osterman, A.L., et al. (2012). Functional specialization in proline biosynthesis of melanoma. PLoS One *7*, e45190.

Eales, K.L., Hollinshead, K.E., and Tennant, D.A. (2016). Hypoxia and metabolic adaptation of cancer cells. Oncogenesis *5*, e190.

Elia, I., Broekaert, D., Christen, S., Boon, R., Radaelli, E., Orth, M.F., Verfaillie, C., Grunewald, T.G.P., and Fendt, S.M. (2017). Proline metabolism supports metastasis formation and could be inhibited to selectively target metastasizing cancer cells. Nat. Commun. *8*, 15267.

Frezza, C., Zheng, L., Tennant, D.A., Papkovsky, D.B., Hedley, B.A., Kalna, G., Watson, D.G., and Gottlieb, E. (2011). Metabolic profiling of hypoxic cells revealed a catabolic signature required for cell survival. PLoS One *6*, e24411.

Harris, A.L. (2002). Hypoxia—a key regulatory factor in tumour growth. Nat. Rev. Cancer *2*, 38–47.

Hollinshead, K.E.R., Munford, H., Eales, K.L., Bardella, C., Li, C., Escribano-Gonzalez, C., Thakker, A., Nonnenmacher, Y., Kluckova, K., Jeeves, M., et al. (2018). Oncogenic IDH1 mutations promote enhanced proline synthesis through PYCR1 to support the maintenance of mitochondrial redox homeostasis. Cell Rep. *22*, 3107–3114.

Ivascu, A., and Kubbies, M. (2006). Rapid generation of single-tumor spheroids for high-throughput cell function and toxicity analysis. J. Biomol. Screen *11*, 922–932.

Krishnan, N., Dickman, M.B., and Becker, D.F. (2008). Proline modulates the intracellular redox environment and protects mammalian cells against oxidative stress. Free Radic. Biol. Med. *44*, 671–681.

LaNoue, K.F., Walajtys, E.I., and Williamson, J.R. (1973). Regulation of glutamate metabolism and interactions with the citric acid cycle in rat heart mitochondria. J. Biol. Chem. *248*, 7171–7183.

Liu, M., Wang, Y., Yang, C., Ruan, Y., Bai, C., Chu, Q., Cui, Y., Chen, C., Ying, G., and Li, B. (2020). Inhibiting both proline biosynthesis and lipogenesis synergistically suppresses tumor growth. J. Exp. Med. *217*, e20191226.

Loayza-Puch, F., Rooijers, K., Buil, L.C., Zijlstra, J., Oude Vrielink, J.F., Lopes, R., Ugalde, A.P., van Breugel, P., Hofland, I., Wesseling, J., et al. (2016). Tumour-specific proline vulnerability uncovered by differential ribosome codon reading. Nature *530*, 490–494.

Luengo, A., Li, Z., Gui, D.Y., Sullivan, L.B., Zagorulya, M., Do, B.T., Ferreira, R., Naamati, A., Ali, A., Lewis, C.A., et al. (2021). Increased demand for NAD(+) relative to ATP drives aerobic glycolysis. Mol. Cell *81*, 691–707.e696.

Markert, C.L. (1984). Lactate dehydrogenase. Biochemistry and function of lactate dehydrogenase. Cell Biochem. Funct. *2*, 131–134.

McGregor, G.H., Campbell, A.D., Fey, S.K., Tumanov, S., Sumpton, D., Blanco, G.R., Mackay, G., Nixon, C., Vazquez, A., Sansom, O.J., et al. (2020). Targeting the metabolic response to statin-mediated oxidative stress produces a synergistic antitumor response. Cancer Res. *80*, 175–188.

Milne, K., Sun, J., Zaal, E.A., Mowat, J., Celie, P.H.N., Fish, A., Berkers, C.R., Forlani, G., Loayza-Puch, F., Jamieson, C., et al. (2019). A fragment-like approach to PYCR1 inhibition. Bioorg. Med. Chem. Lett. *29*, 2626–2631.

Phang, J.M. (2019). Proline metabolism in cell regulation and cancer biology: recent advances and hypotheses. Antioxid. Redox Signal. *30*, 635–649.

Phang, J.M., Liu, W., Hancock, C., and Christian, K.J. (2012). The proline regulatory axis and cancer. Front. Oncol. *2*, 60.

Phang, J.M., Liu, W., and Zabirnyk, O. (2010). Proline metabolism and microenvironmental stress. Annu. Rev. Nutr. *30*, 441–463.

Phang, J.M., Pandhare, J., and Liu, Y. (2008). The metabolism of proline as microenvironmental stress substrate. J. Nutr. *138*, 2008S–2015S.

Sahu, N., Dela Cruz, D., Gao, M., Sandoval, W., Haverty, P.M., Liu, J., Stephan, J.P., Haley, B., Classon, M., Hatzivassiliou, G., et al. (2016). Proline starvation induces unresolved ER stress and hinders mTORC1-dependent tumorigenesis. Cell Metab. *24*, 753–761.

Schworer, S., Berisa, M., Violante, S., Qin, W., Zhu, J., Hendrickson, R.C., Cross, J.R., and Thompson, C.B. (2020). Proline biosynthesis is a vent for TGFbeta-induced mitochondrial redox stress. EMBO J. *39*, e103334.

Scott, G.K., Yau, C., Becker, B.C., Khateeb, S., Mahoney, S., Jensen, M.B., Hann, B., Cowen, B.J., Pegan, S.D., and Benz, C.C. (2019). Targeting mitochondrial proline dehydrogenase with a suicide inhibitor to exploit synthetic lethal interactions with p53 upregulation and glutaminase inhibition. Mol. Cancer Ther. *18*, 1374–1385.

Sullivan, L.B., Gui, D.Y., Hosios, A.M., Bush, L.N., Freinkman, E., and Vander Heiden, M.G. (2015). Supporting aspartate biosynthesis is an essential function of respiration in proliferating cells. Cell *162*, 552–563.

Tran, D.H., Kesavan, R., Rion, H., Hoseini Soflaee, M., Solmonson, A., Bezwada, D., Vu, H.S., Cai, F., Phillips, J.A., DeBerardinis, R.J., et al. (2021). Mitochondrial NADP$^+$ is essential for proline biosynthesis during cell growth. Nat. Med. *3*, 571–585.

Vettore, L.A., Westbrook, R.L., and Tennant, D.A. (2021). Proline metabolism and redox; maintaining a balance in health and disease. Amino Acids *53*, 1779–1788.

Wykoff, C.C., Beasley, N., Watson, P.H., Campo, L., Chia, S.K., English, R., Pastorek, J., Sly, W.S., Ratcliffe, P., and Harris, A.L. (2001). Expression of the hypoxia-inducible and tumor-associated carbonic anhydrases in ductal carcinoma in situ of the breast. Am. J. Pathol. *158*, 1011–1019.

Zhu, J., Schworer, S., Berisa, M., Kyung, Y.J., Ryu, K.W., Yi, J., Jiang, X., Cross, J.R., and Thompson, C.B. (2021). Mitochondrial NADP(H) generation is essential for proline biosynthesis. Science *372*, 968–972.

STAR★METHODS

KEY RESOURCES TABLE

REAGENT or RESOURCE	SOURCE	IDENTIFIER
Antibodies		
Carbonic Anhydrase IX	Novus Biologicals	Cat#NB100-417; RRID:AB_10003398
Ki67	Cell Signaling	Cat#12202; RRID:AB_2620142
Actin	Sigma	Cat#A4700; RRID:AB_476730
PYCR1	Proteintech	Cat#13108-1-AP; RRID:AB_2174878
PYCR2	Proteintech	Cat#17146-1-AP; RRID:AB_2253344
HIF1α	BD Biosciences	Cat#610959; RRID:AB_398272
Anti-Rabbit-HRP	Cell Signalling	Cat#7074S; RRID:AB_2099233
Anti-Mouse-HRP	Cell Signalling	Cat#7076S; RRID:AB_330924
PYCR1	Cell Signalling	Cat#37635; RRID:AB_2904491
Ki67	Agilent	Cat#M7240; RRID:AB_2142367
CD31	Agilent	Cat#M082301-2; RRID:AB_2904492
Carbonic Anhydrase 9	Absolute Antibody	Cat#M75; RRID:AB_2904493
GLUT1	Abcam	Cat#ab652; RRID:AB_305540
Critical commercial assays		
NAD^+:NADH assay kit	Biovision	Cat#K337
GSH:GSSG assay kit	Promega	Cat#V6612
Experimental models: Cell lines		
HCC1806	ATCC	CRL-2335; RRID:CVCL_1258
SUM159PT	BioIVT	HUMANSUM-0003006
SUM159PT $PYCR1^{-/-}$	Professor Reuven Agami	N/A
MDA-MB-231	ATCC	HTB-26; RRID:CVCL_0062
HS5	ATCC	CRL-11882; RRID:CVCL_3720
ONS-76	Accegen	ABC-TC0875
Doxycycline-inducible shPYCR1 HCC1806	This paper	N/A
Oligonucleotides		
PYCR1 siRNA	Dharmacon	Cat#J-012349-06-0005
PYCR2 siRNA	Dharmacon	Cat#J-016646-06-0005
Non-targeting siRNA	Dharmacon	Cat#D-001810-10-05
shERWOOD UltraMiR Lentiviral inducible pZip target gene set for PYCR1	Transomic	Cat# TLHSU2300-5831
Software and algorithms		
MATLAB	Mathworks	N/A
GraphPad Prism	GraphPad Software	N/A
Adobe Illustrator	Adobe	N/A
Deposited data		
Metabolic data	N/A	Mendeley Data: https://doi.org/10.17632/vbjp8xs9k6.1

RESOURCE AVAILABILITY

Lead contact

Further information and requests for resources and/or reagents should be directed to and will be fulfilled by Professor Daniel A Tennant (D.Tennant@bham.ac.uk)

Materials availability

Materials generated in this study are available from the lead contact.

Data and code availability

- Metabolic tracing data have been deposited at Mendeley and are publicly available as of the date of publication. DOIs are listed in the key resources table.
- This paper does not report any original code
- Any additional information required to reanalyze the data reporting in this paper is available from the lead author on request.

EXPERIMENTAL MODEL AND SUBJECT DETAILS

Cell culture

HCC1806, MDA-MB-231 and HS5 cell lines were purchased from ATCC, SUM159-PT from BioIVT and ONS-76 from Accegen. The SUM159PT PYCR1$^{-/-}$ and paired control were previously reported (Loayza-Puch et al., 2016). All cell lines were passaged when they reached 70%–80% confluence, using 0.05% Trypsin (Gibco, 15400-054) for dissociation and 1x phosphate buffered saline (PBS) for washing. MDA-MB-231, HCC1806 and HS5 cells were maintained in RPMI (Sigma-Aldrich, R8758) with 10% FBS. SUM159-PT cells were growth in Dulbecco's Modified Eagle's Medium (DMEM) Nutrient Mixture F-12 Ham (Sigma, D8062), with 5% FBS, 5 mg/ml insulin (Sigma, I6634) and 1 μg/ml hydrocortisone (Sigma, H0888). ONS-76 cell lines were grown in DMEM Nutrient Mixture F-12 Ham (Sigma, D8062), with 10% FBS. Doxycycline-inducible shPYCR1 expressing HCC1806 cell line was established using shERWOOD UltraMiR Lentiviral inducible pZip target gene set for PYCR1 (Transomic, TLHSU2300-5831).

In vivo model

Fresh and sterile doxycycline was used in these experiments. 100 μl of matrigel containing 2 × 10^6 HCC-1806 doxycycline –shControl (shC) or -inducible shPYCR1 cells were established into the mammary fat pad of CD1 nude female mice aged 5–6 weeks. Where shown, mice were given doxycycline when tumor growth reached average tumor volume 50 mm^3 (single dose study) or 75 mm^3 (chronic dosing study). Single dose: mice (7 mice per group of shNT or shPYCR1) were administered one dose of oral gavage of doxycycline (25 mg/kg). Adverse effects on animal welfare was observed in the shPYCR1 group only, with one mortality and remaining six mice displayed moderate changes in welfare, including significantly reduced mobility and decreased body temperature. Chronic dosing: mice were administered oral gavage of doxycycline (10 mg/kg) every two days rota (one day treat and one day rest) until endpoint. No changes in animal welfare was observed in either group (of shC or shPYCR1). Endpoint procedures: Single dose: animals were culled the day after dosing, and tumors sectioned for both flash-freezing and fixation for downstream analysis. Chronic dosing: when tumors attained endpoint size permitted mice were injected with $^{13}C_5$-glutamine (3 × 200 μl [7.2 mg] each 36.2 mg/ml stock solution at 15 min intervals [total = 142 μmol] 45 minutes before cull. Pimonidazole (2 mg/kg) and a 647-Tomato lectin (1 mg/kg)/Hoescht (2 mg/kg) mix was given intravenous 5 min before visualization on IVIS system for fluorescence imaging of perfused tumors and mice culled. Tumors were excised, sectioned, and samples flash-frozen and fixed for downstream analysis. Blood samples were also collected by cardiac puncture when culling mice.

METHOD DETAILS

Spheroids

Cells were trypsinized, counted, and either plated in 6 well plates coated with 0.5% agarose in DMEM or on low adherence U-bottomed 96 well plates (Thermo, 174925) and then centrifuged for 5 minutes at 500 × g. For proline supplementation experiments, spheroids were seeded in DMEM flux media (without phenol red, glutamine, and glucose) ((Gibco, A14430), with or without 1mM proline. For all other experiments spheroids were left to form for at least 72 hours before investigation. For staining, spheroids were grown for 7 days before fixing in neutral buffered formalin overnight, and transfer to 70% ethanol for storage. The solution containing spheroids was transferred onto the center of a sheet of biopsy paper (CellPath, UK) before processing for histology. All staining was performed on 4 μm formalin fixed paraffin embedded (FFPE) sections. Sections for Haematoxylin & Eosin staining were stained using a Leica ST5020 autostainer. Carbonic Anhydrase XI (NB100-417, Novus Biologicals), Caspase 3 (9661, Cell Signaling) and Ki67 (12202, Cell Signaling) antibodies were stained on a Leica Bond Rx autostainer. FFPE sections were loaded onto the autostainer and underwent dewaxing (Leica, AR9222) and epitope retrieval on board. Carbonic Anhydrase XI, Caspase 3 and Ki67 was retrieved using ER2 buffer (Leica, AR9640). All sections were retrieved at 95°C for 20 minutes. After retrieval sections were rinsed with wash buffer (Leica, AR9590) before peroxidase block was performed for 5 minutes using an Intense R kit (Leica, DS9263). Sections were rinsed with wash buffer. After rinsing with wash buffer the antibodies were applied at previously optimized dilutions (Carbonic Anhydrase XI, 1/250; Caspase 3, 1/500; Ki67, 1/1000) for 30 minutes before washing with buffer and then application of appropriate secondary antibody (Carbonic Anhydrase XI and Ki67 – Rabbit EnVision, Agilent, K4003) for 30 minutes. Sections were rinsed with wash buffer before being visualized using DAB in the Intense R kit. The sections were counterstained with Haematoxylin in the Intense R kit

before coverslipping the sections with DPX mountant (CellPath, SEA-1300-00A). Images were captured from the stained sections using a Leica Aperio AT2 slide scanner.

Metabolic tracing

For tracing experiments, cells were plated to be 70% confluent after 24 hours. Media was then changed to flux media (without phenol red, glutamine, and glucose) ((Gibco, A14430)), depending on cell line, with 10 mM glucose and 2 mM glutamine (either one universally labelled, depending on experiment). Cells were transferred into hypoxic conditions (Don Whitley H35 Hypoxystation). After 24 hours, 100 μl of media was removed for extraction. The rest of the media was removed and wells were washed twice with ice cold saline, 500 μl MeOH was added, with 200 μl D_6-glutaric acid (2.5 mg/ml) (CDN isotopes, D-5227). Cells were scraped and transferred to a cold Eppendorf with 500 μl ice-cold H_2O and the same volume of chloroform (pre-chilled to −20°C). After shaking on ice for 15 minutes and centrifugation, the polar phase was transferred to another tube for derivatization, which was dried with centrifugation at 45°C. Spheroids grown in U-bottom 96 well plates were collected and washed with PBS before transfer to flux media with ^{13}C-[U]-glutamine in agarose-coated 12 well plates and incubated for 48 hours. Spheroids were collected and washed in ice cold saline, transferred to ice cold MeOH and homogenized (2 × 30 seconds) using a Precellys 24 tissue homogenizer (Bertin Instruments). Metabolite extraction was performed as previously described in cells. Data were normalized to total ion abundance.

Glucose and lactate consumption

Media was changed to flux DMEM (without phenol red, glutamine, and glucose) (Gibco, A14430) with 10 mM glucose and 2 mM glutamine added. Glucose and lactate measurements were taking using a Nova Biomedical Stat Profile Prime CCS Analyzer and normalized to total protein.

Bicinchoninic acid (BCA) protein assay

Spheroids were collected and washed with PBS, before sonication for 10 minutes in 0.2 M NaOH, and boiling. The Pierce BCA Protein Assay Kit (Thermo Scientific, 23225) was used according to the manufacturers protocol.

Derivatization and GCMS

Dried down extracts are derivatized using a two-step protocol. Samples are first treated with 2% methoxamine in pyridine (40 μl, 1 h at 60°C), followed by addition of N-(tert-butyldimethylsilyl)-N-methyl-trifluoroacetamide, with 1% tert-butyldimethylchlorosilan (50 μl, 1 h at 60°C). Samples are transferred to glass vials for GC-MS analysis using an Agilent 8890 GC and 5977B MSD system. 1 μL of sample was injected in splitless mode with helium carrier gas at a rate of 1.0 mL.min^{-1}. Initial GC oven temperature was held at 100°C for 1 minute before ramping to 160°C at a rate of 10°C.min^{-1}, followed by a ramp to 200°C at a rate of 5°C.min^{-1} and a final ramp to 320°C at a rate of 10°C.min^{-1} with a 5 minute hold. Compound detection was carried out in scan mode. Total ion counts of each metabolite were normalized to the internal standard D^6-Glutaric acid.

Western blotting

Cells were washed once with 1 x PBS and scraped into 1x Laemmli buffer (Sigma, 2301-1VL). Samples were heated for 10 minutes at 100°C and briefly centrifuged, then vortexed. Samples were run at 150V through a 10% polyacrylamide gel. Protein was transferred to a nitrocellulose membrane using an Invitrogen iBlot. Membranes were blocked with 5% milk (Marvel) in PBS for 1 hour, before overnight incubation at 4°C with the primary antibody in 1% milk; actin (1:4000 dilution, Sigma, A4700), PYCR1 (1:5000 dilution, Proteintech, 13108-1-AP), PYCR2 (1:2000 dilution, Proteintech, 17146-1-AP), HIF1α (1:500 dilution, BD Biosciences, 610959). PBS-Tween was used for membrane washing before incubation with the secondary antibody at room temperature for 1 hour: Anti-Rabbit-HRP (Cell Signalling, 7074S) or Anti-Mouse-HRP (Cell Signalling, 7076S). After another washing step, EZ-ECL (Biological Industries, 20-500-120) was added to the membrane before imaging using a BioRed ChemiDoc Imaging System. Densitometry was performed using ImageJ-Fiji, values were normalized to loading controls. For siRNA blots, values were also normalized to the non-target.

Cell number and GCMS data normalization values

To assess cell number, a Sulforhodamine B (SRB) assay was used as previously described. 100 μL of 20% trichloracetic acid was added to each well and incubated at 4°C for 30 minutes. Wells were washed with tap water three times and allowed to dry. 0.4% Sulforhodamine B (Sigma, 230162) was added to cover the surface of the wells and incubated for 10 minutes at room temperature. Wells were washed with 1% acetic acid four times and again allowed to dry. The stain was dissolved in 50 mM tris at pH8.8 and absorbance was measured at 510 nm.

Oxygen consumption

Trypsinized cells were resuspended in media as above and loaded in an Oxygraph-2k (Oroboros instruments) chamber. After closing the chambers and recording routine respiration, oligomycin (2.5 μM) was added to inhibit ATP synthase. Measurements of the non-phosphorylating electron transfer system (ETS) capacity were obtained through stepwise (0.5 μM) titration of the uncoupler, carbonyl cyanide 4-(trifluoromethoxy)phenylhydrazone (FCCP). Respiration was inhibited by addition of rotenone (0.5 μM) and antimycin A (2.5 μM) at the end of the experiment.

GSH:GSSG assay

Cells were plated in 6 well plates to be 70% confluent for transfection. Transfection with siRNA at 50nM, either: siNT (Dharmacon, D-001810-10-05), siPYCR1 (Dharmacon, J-012349-06-0005) or siPYCR2 (Dharmacon, J-016646-06-0005) using Dharmafect 1 (Dharmacon, T-2001-03). After 24 hours, transfected cells were re-plated in white, clear bottom 96 well plates (Corning, 3903), and transferred to hypoxia. After 24 hours GSH:GSSH was measured using the Promega GSH:GSSG-Glo assay kit (Promega, V6612) according to manufacturer's protocol.

NAD^+:NADH assay

Cells were plated to be 70% confluent for assay. Cells were harvested and the assay was carried out according to the manufacturer's protocol (Biovision kit K337).

Immunohistochemistry

To summarize staining method the slides were firstly dewaxed in histoclear, rehydrated using decreasing concentrations of ethanol to tap water. A standard haematoxylin and eosin protocol was followed to assess the morphology and the amount of necrosis on xenografts. For immunohistochemistry staining antigen retrieval performed in pH 6 for all antibodies and, slides were stained using the FLEX staining protocol and reagents (Agilent). Endogenous peroxidase activity was blocked before slides were stained with primary antibodies: proliferation marker Ki67 (mouse, MIBI, 1:50 M7240 Agilent), endothelial marker CD31 (rabbit, JC70, Agilent), hypoxia marker CA9 (mouse, M75, Absolute antibody, 1:100), PYCR1 (rabbit, E7P3I, cell signaling), GLUT1 (rabbit, ab652, Abcam, 1:100). Slides incubated for 2h at room temperature. Slides were washed in Flex buffer before then being incubated with the Flex anti-rabbit/mouse secondary antibody for 30 minutes at room temperature and washed in Flex buffer. 3,3′-Diaminobenzidine (Flex-DAB) was applied to the sections for 10 minutes. The slides were counterstained by immersing in Flex-hematoxylin solution for 5 min, washed, and air-dried before mounted with mounting medium (Sigma). Secondary-only control staining was done routinely, these were negative. Expression of markers and viable/necrotic areas was quantified on whole sections quantitatively by using the Visiopharm Integrator System. HDAB-DAB color deconvolution band is used to detect positively stained cells. Threshold classification is used to identified necrosis and living regions and thus identify number of positive staining within these regions. Appropriate thresholds levels are checked against control xenografts staining before being set and the xenografts from all groups are then analyzed. Quantification based on intensity of staining (value calculated from pixilation of DAB, corresponding to stain intensity from no staining, weak to strong staining) and percentage of coverage (value calculated from DAB positive total area expressed relative to the total area of tissue).

QUANTIFICATION AND STATISTICAL ANALYSIS

GCMS data were analyzed using Agilent Mass Hunter software for real time analysis of data quality, before conversion to .CDF format and analysis with in-house MATLAB scripts. Graphs and statistical analysis were performed using GraphPad Prism 9. Where a 2-sample analysis is shown, results are from a Mann-Whitney test, while with >2 samples, results of a Kruskal-Wallis test are shown with Dunn's multiple comparison post hoc. Sample numbers are included in figure legends. To perform a statistical comparison of the xenograft growth curves was performed through first fitting a single exponential curve (Figures S5B–S5E), the integral of which was used to generate a linear regression for each xenograft (Figures S5F–S5I). These equations were then used to apply a Mann-Whitney test.

Cell Reports, Volume *38*

Supplemental information

Proline synthesis through PYCR1 is required to support cancer cell proliferation and survival in oxygen-limiting conditions

Rebecca L. Westbrook, Esther Bridges, Jennie Roberts, Cristina Escribano-Gonzalez, Katherine L. Eales, Lisa A. Vettore, Paul D. Walker, Elias Vera-Siguenza, Himani Rana, Federica Cuozzo, Kattri-Liis Eskla, Hans Vellama, Abeer Shaaban, Colin Nixon, Hendrik Luuk, Gareth G. Lavery, David J. Hodson, Adrian L. Harris, and Daniel A. Tennant

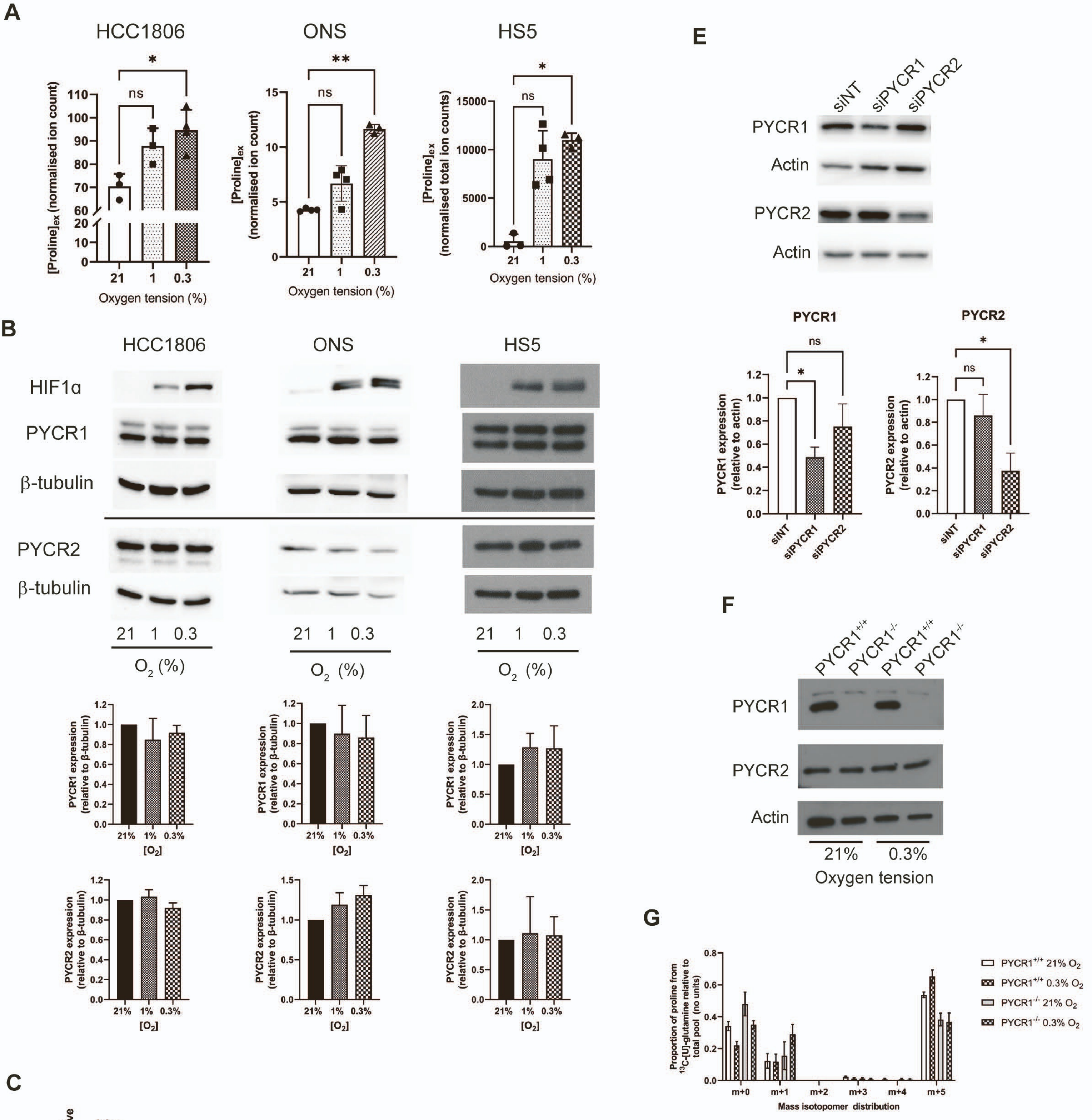

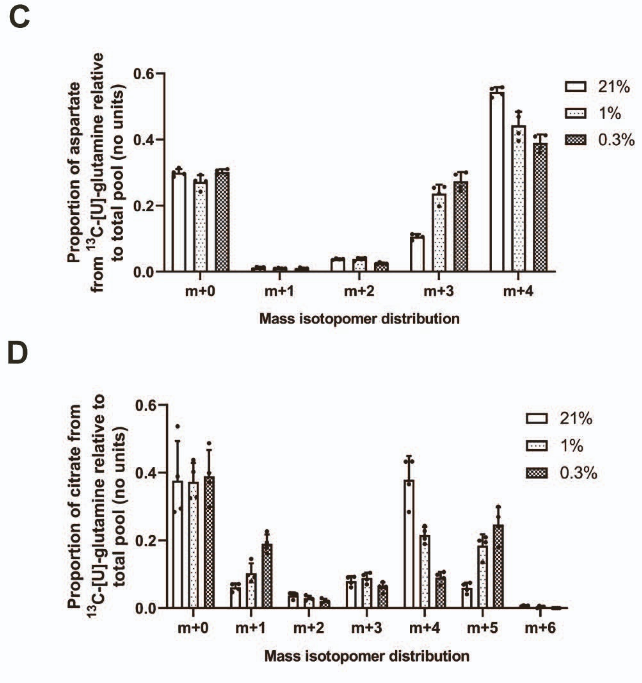

Supplementary Figure 1. Glutamine-derived proline export is increased in hypoxia without increased expression PYCR1 or PYCR2. Related to Figure 1. A: Extracellular proline is significantly increased in hypoxia (0.3% O_2) compared to normoxia (21% O_2) in the HCC1806, ONS and HS5 cell lines. *, $p<0.05$; **, $p<0.01$. **B:** Expression of PYCR1 and PYCR2 does not change with oxygen tension in HCC1806, ONS or HS5 cell lines. Densitometry shown is PYCR1 or 2 normalised to b-tubulin, SD of three independent blots shown. **C** and **D**: As oxygen decreases, the expected decrease in TCA cycle activity (m+4 aspartate [**C**] and citrate [**D**]) and relative increase in reductive carboxylation (m+5 citrate [**D**] and m+3 aspartate [**C**]) is observed in SUM159PT cells. **E:** Protein expression of PYCR1 and PYCR2 with siRNA knockdown of each isoform in SUM159PT cells. Actin as loading control. Densitometry shown below blots for PYCR1 and PYCR2 showing significant knockdown of the relevant isozyme. Statistical analysis shown is result of Kruskal-Wallis ANOVA with Dunn's post hoc test. *, $p<0.05$. **F:** Expression of PYCR1 and PYCR2 in the SUM159PT PYCR1$^{-/-}$ cell line in 21% and 0.3% oxygen. **G:** Mass isotopomer distribution of ^{13}C-[U]-glutamine incorporation into intracellular proline relative to the total pool in SUM159PT PYCR1$^{+/+}$ and PYCR1$^{-/-}$ cells. All metabolic data presented is mean of n=3 ± SD.

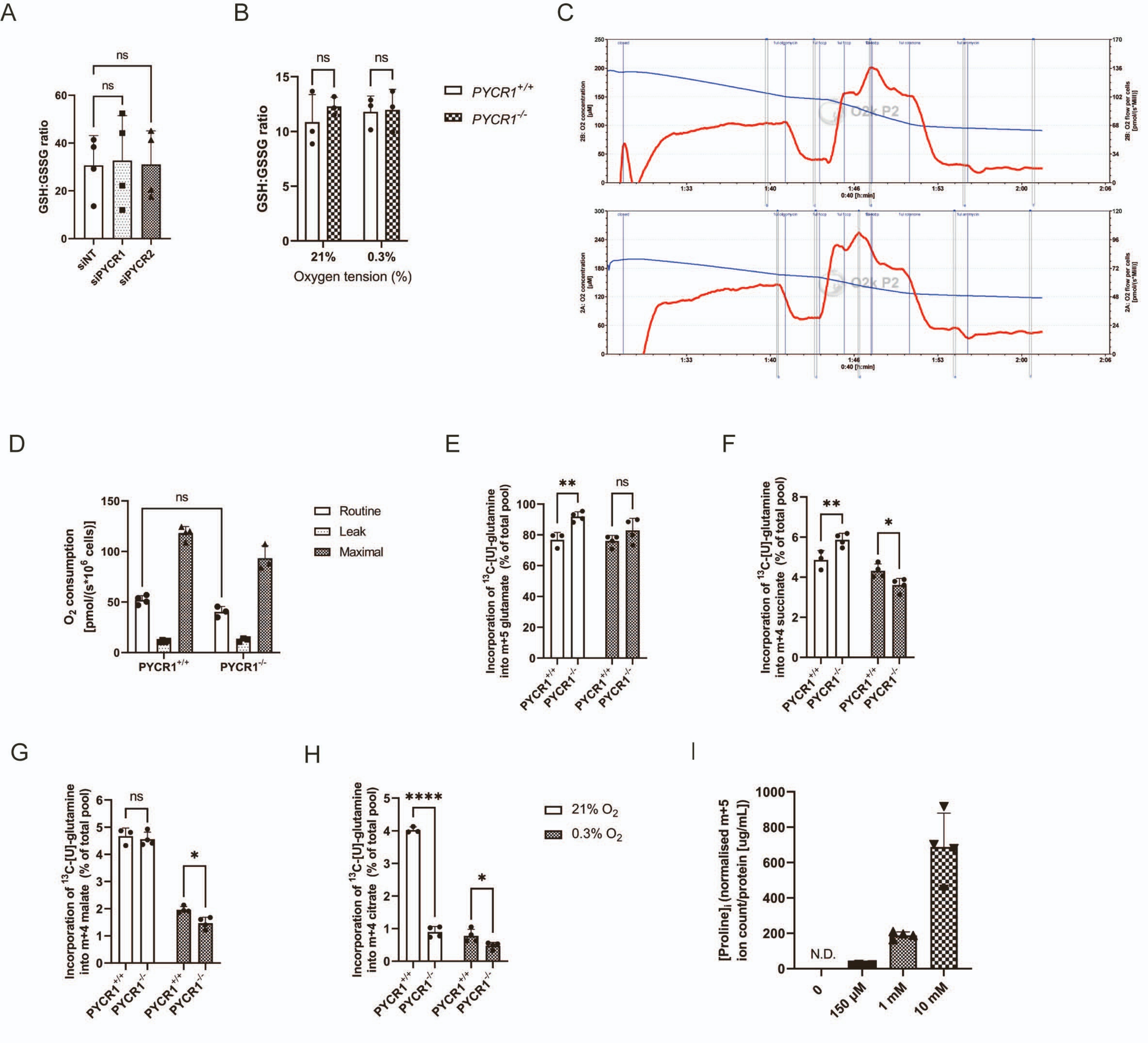

Supplementary Figure 2. Loss of PYCR1 in 2D cell culture. A: siPYCR1 or 2 does not change GSH:GSSG ratio in the SUM159PT cell line. Related to Figure 2. B: PYCR1$^{-/-}$ cells do not exhibit an alteration in the GSH:GSSG ratio in normoxia or hypoxia. **C:** Representative oxygraph traces of PYCR1$^{+/+}$ (top) and PYCR1$^{-/-}$ (bottom) cells. **D:** There is no significant difference in oxygen consumption in PYCR1$^{-/-}$ cells compared to PYCR1$^{+/+}$. Steady-state levels of glutamate (**E**), succinate (**F**), malate (**G**) and citrate (**H**). *, p<0.05; **, p<0.01; ****, p<0.0001. **I:** Abundance of intracellular ^{13}C-[U]-proline in SUM159PT cells incubated with increasing concentrations. All data presented as mean ± SD

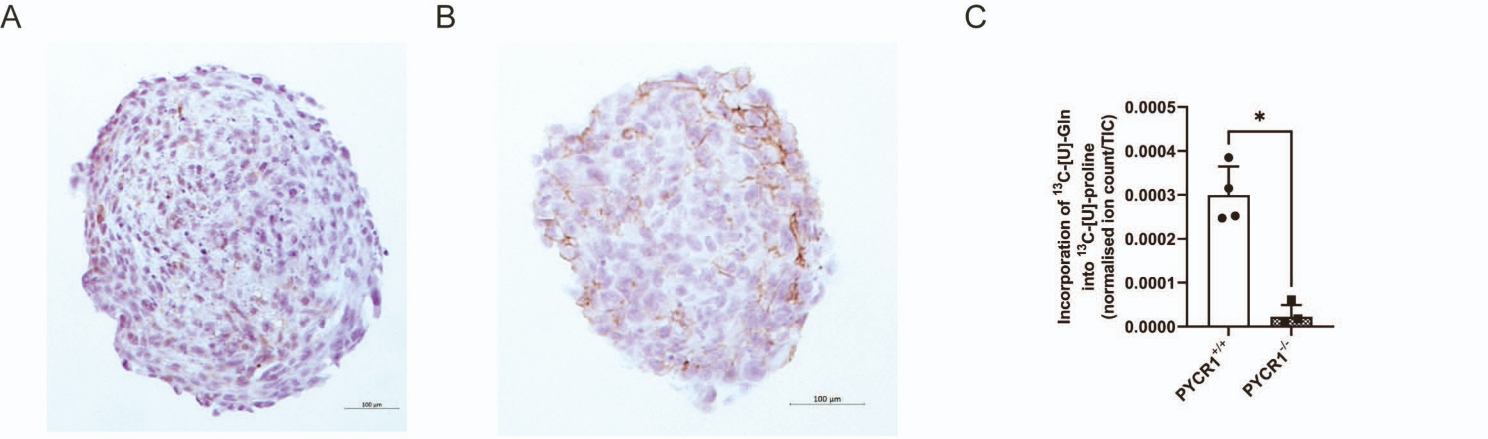

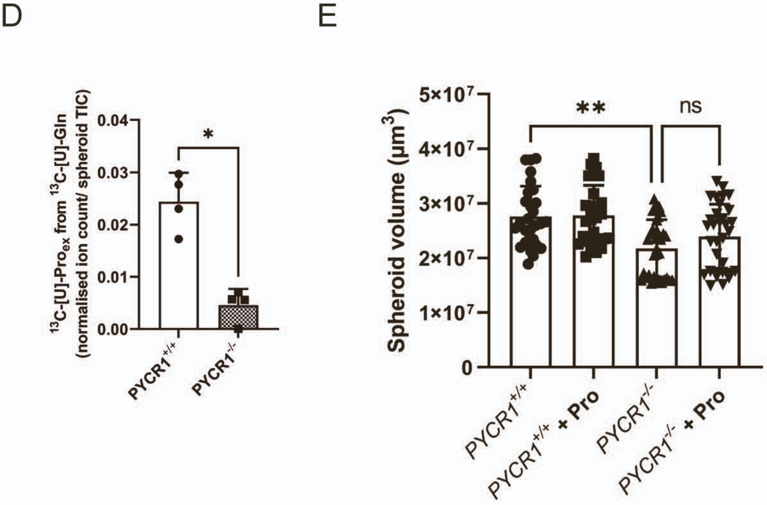

Supplementary Figure 3: Loss of PYCR1 in a 3D cell culture model. Related to Figure 3. A: Image of a representative SUM159PT spheroid stained with the hypoxia marker, pimonidazole, showing diffuse areas of hypoxia. **B:** Image showing staining of the hypoxia-induced glucose transporter, GLUT1 in SUM159PT spheroid. **C:** PYCR1$^{-/-}$ spheroids synthesise less ^{13}C-[U]-proline from ^{13}C-[U]-glutamine. *, $p<0.05$. This is reflected in reduced excretion of proline into the media (**D**), consistent with data from 2D culture. *, $p<0.05$. **E:** PYCR1$^{-/-}$ spheroid growth is not rescued by exogenous proline supplementation. All data presented as mean ± SD

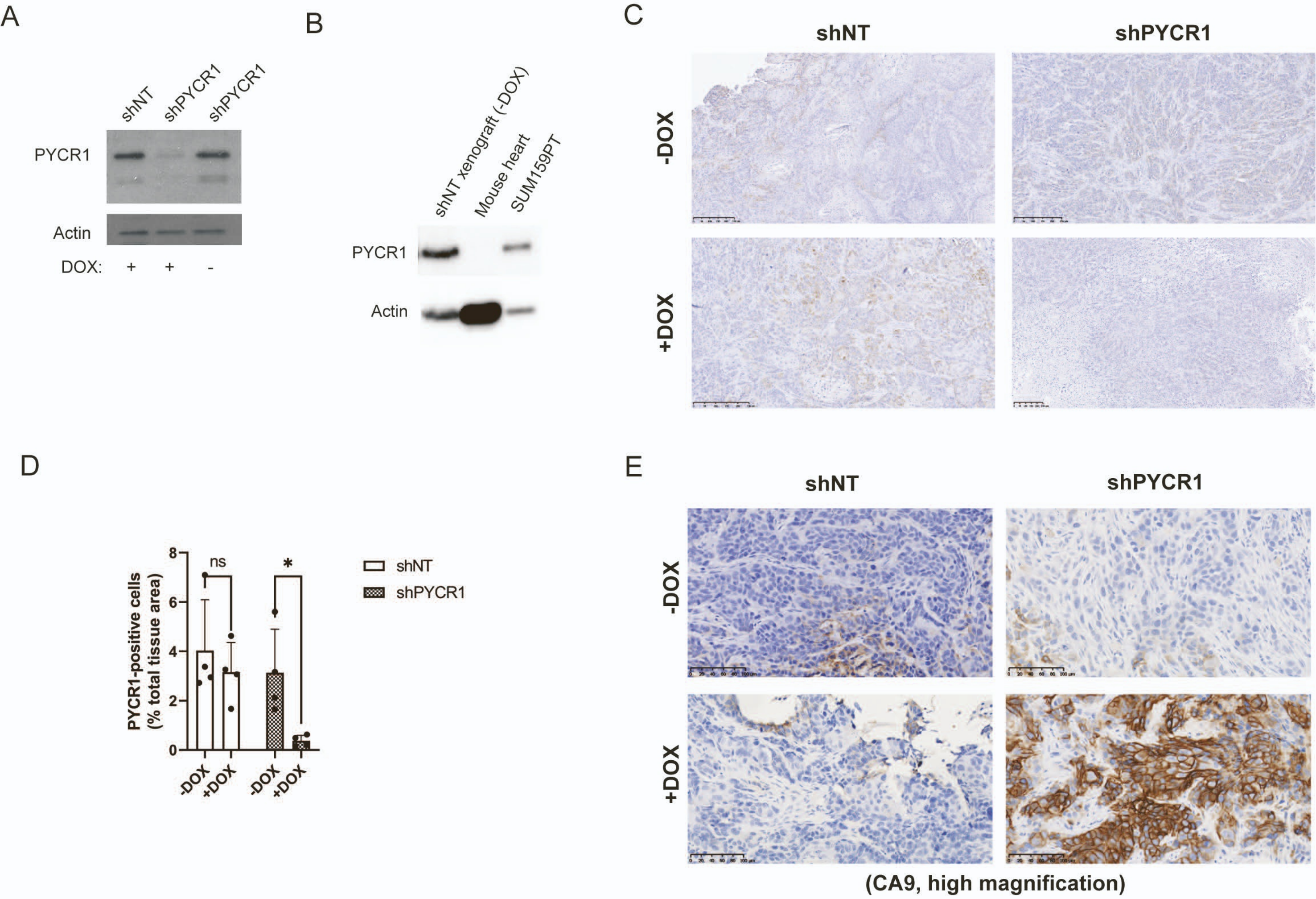

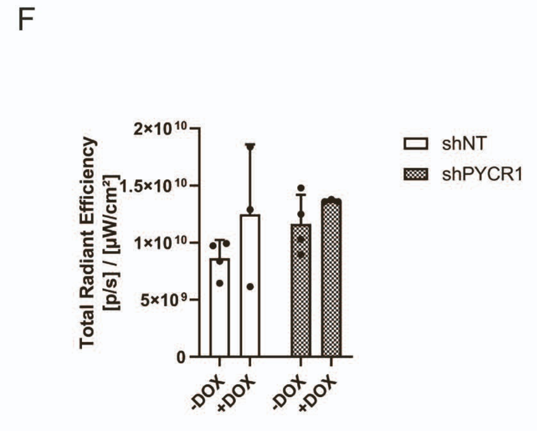

Supplementary Figure 4: Acute inducible PYCR1 knockdown in vivo xenografts. Related to Figure 4. A: Doxycycline elicits knockdown of PYCR1 in shPYCR1 cell model. **B:** Antibody against PYCR1 used to stain xenograft tissue (C) is specific for human protein. **C:** Representative images showing that PYCR1 expression in the tumour cells of the xenograft is reduced after DOX treatment. **D:** Quantification of PYCR1 staining in all tumours showing a reduction in PYCR1 expression after DOX treatment. *, $p<0.05$. **E:** Representative images at high magnification of CA9 staining in xenografts from the groups indicated, showing significantly increased hypoxia only in those tumours with shPYCR1+DOX. **F:** No significant differences in vascularity (measured through total radiant efficiency) were observed between groups (Figure 4). All quantitative data presented as mean ± SD

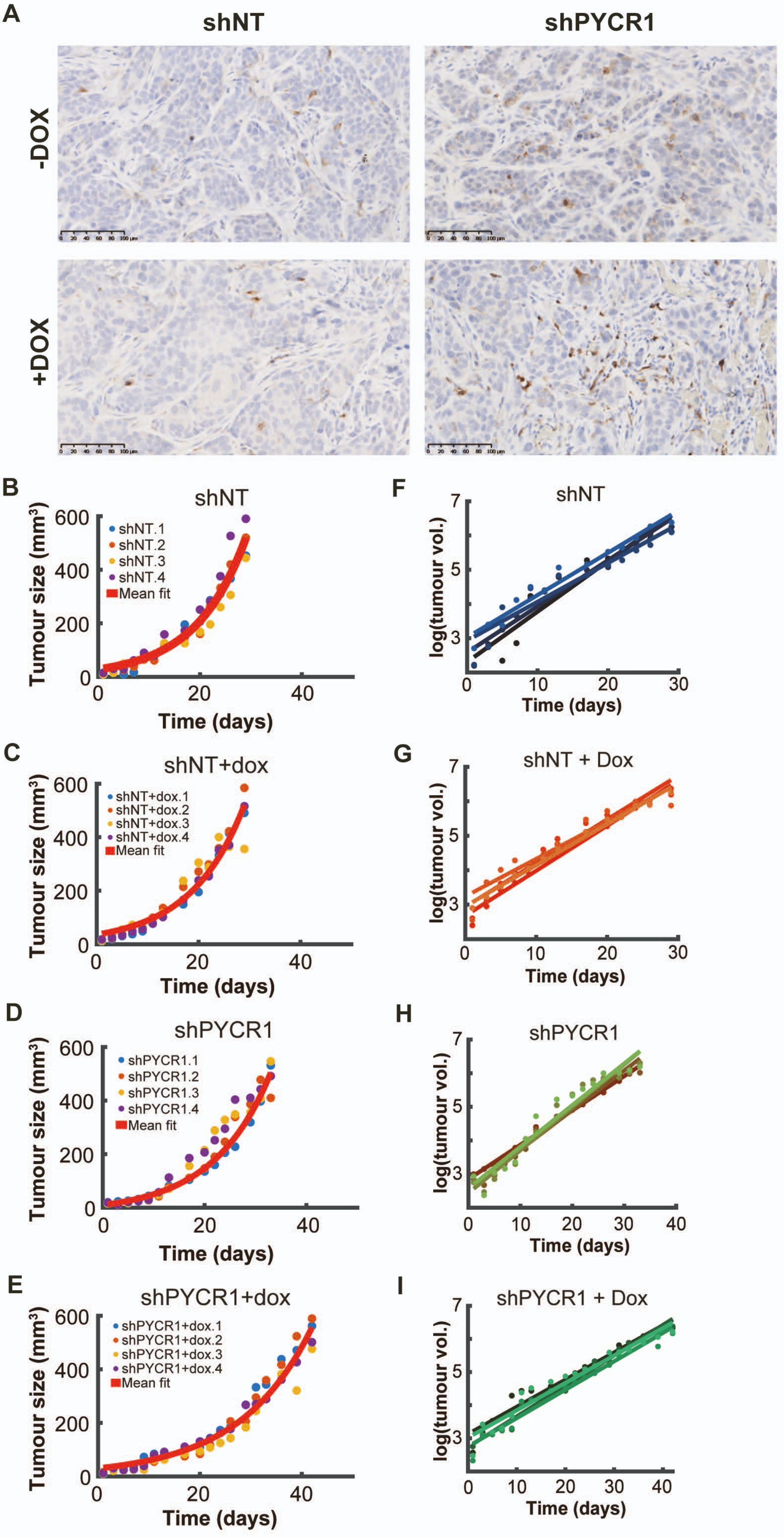

Supplementary Figure 5: Xenograft phenotype and xenograft growth curve fitting. Related to Figure 5. A: Representative higher magnification image of xenograft tumours stained for cleaved caspase-3. Scale bar = 100 mm. **B-E:** Exponential model fit of xenograft growth curves. **F-I:** Linear regression of log-transformed fitted curves B-E, allowing for appropriate statistical analysis (results shown in Figure 5F).
